# SREBP Coordinates Iron and Ergosterol Homeostasis to Mediate Triazole Drug and Hypoxia Responses in the Human Fungal Pathogen *Aspergillus fumigatus*

**DOI:** 10.1371/journal.pgen.1002374

**Published:** 2011-12-01

**Authors:** Michael Blatzer, Bridget M. Barker, Sven D. Willger, Nicola Beckmann, Sara J. Blosser, Elizabeth J. Cornish, Aurelien Mazurie, Nora Grahl, Hubertus Haas, Robert A. Cramer

**Affiliations:** 1 Division of Molecular Biology/Biocenter, Innsbruck Medical University, Innsbruck, Austria; 2 Department of Immunology and Infectious Diseases, Montana State University, Bozeman, Montana, United States of America; 3 Bioinformatics Core and Department of Microbiology, Montana State University, Bozeman, Montana, United States of America; University of California San Francisco, United States of America

## Abstract

Sterol regulatory element binding proteins (SREBPs) are a class of basic helix-loop-helix transcription factors that regulate diverse cellular responses in eukaryotes. Adding to the recognized importance of SREBPs in human health, SREBPs in the human fungal pathogens *Cryptococcus neoformans* and *Aspergillus fumigatus* are required for fungal virulence and susceptibility to triazole antifungal drugs. To date, the exact mechanism(s) behind the role of SREBP in these observed phenotypes is not clear. Here, we report that *A. fumigatus* SREBP, SrbA, mediates regulation of iron acquisition in response to hypoxia and low iron conditions. To further define SrbA's role in iron acquisition in relation to previously studied fungal regulators of iron metabolism, SreA and HapX, a series of mutants were generated in the Δ*srbA* background. These data suggest that SrbA is activated independently of SreA and HapX in response to iron limitation, but that HapX mRNA induction is partially dependent on SrbA. Intriguingly, exogenous addition of high iron or genetic deletion of *sreA* in the Δ*srbA* background was able to partially rescue the hypoxia growth, triazole drug susceptibility, and decrease in ergosterol content phenotypes of Δ*srbA*. Thus, we conclude that the fungal SREBP, SrbA, is critical for coordinating genes involved in iron acquisition and ergosterol biosynthesis under hypoxia and low iron conditions found at sites of human fungal infections. These results support a role for SREBP–mediated iron regulation in fungal virulence, and they lay a foundation for further exploration of SREBP's role in iron homeostasis in other eukaryotes.

## Author Summary

Advances in medical technologies over the past several years have led to an increasing population of patients susceptible to fungal infections. Despite the immunocompromised condition of most patients that acquire these infections, the majority are caused by three fungi: *Candida albicans*, *Cryptococcus neoformans*, and *Aspergillus fumigatus*. Of these, *A. fumigatus* is least studied, and the ability of this fungus to cause lethal disease in these patients needs more examination. We previously identified a transcription factor in the sterol-regulatory element binding protein family, SrbA, in this pathogenic mold that is critical for virulence and susceptibility to triazole antifungal drugs. The mechanism by which SrbA mediates these clinically relevant phenotypes is unclear. Here, we discover that SrbA is critical for regulation of iron metabolism, particularly through regulation of siderophore production and uptake. We find that *A. fumigatus* requires iron uptake during the initial phases of adaptation to hypoxic microenvironments and that restoration of iron uptake in the *srbA* null mutant is able to partially restore the hypoxia growth defect and triazole susceptibility of this mutant. Taken together, our results identify a new role for this important fungal SREBP and give new insights into the clinically relevant roles of SrbA.

## Introduction

Fungal pathogens face numerous environmental challenges during growth in mammalian hosts that can determine outcomes of host-pathogen interactions. A major factor in host defense against invading fungi is the sequestration of iron, which prevents in vivo fungal growth [1]. Consequently, most fungal pathogens have evolved mechanisms to obtain iron from their hosts and these mechanisms are established fungal virulence attributes [2], [3], [4], [5], [6], [7], [8]. Intriguingly, an association between responses to iron and oxygen limitation emerged from studies in rodents demonstrating increased iron absorption in response to hypoxia [9]. Moreover, hypoxia is known to increase the expression of transferrin, which increases iron availability to host cells under hypoxic stress [10]. The key transcriptional regulator of mammalian responses to hypoxia, hypoxia inducible factor-1 (HIF), has been found to regulate several genes involved in iron metabolism [11], [12], [13], [14]. Thus, an intimate link exists between cellular responses to low oxygen environments and iron availability in eukaryotes. Yet, mechanisms of regulation of this potential link in human pathogenic fungi are largely unknown.

Previous results strongly suggest that mechanisms of both iron acquisition and hypoxia adaptation are critical for fungi to cause disease in humans. Strains of the human fungal pathogen *Aspergillus fumigatus* that no longer make any iron-sequestering siderophores are fully avirulent, while strains deficient in either extracellular or intracellular siderophore production display attenuated virulence [4], [5], [6]. Regulation of iron acquisition in *A. fumigatus* and other fungi that make siderophores is mediated by two key transcription factors SreA and HapX [2], [15]. Null mutants of SreA display increased siderophore production and as expected remain fully virulent in animal models of invasive pulmonary aspergillosis. Conversely, null mutants of HapX have a reduced ability to produce siderophores and are consequently significantly attenuated in virulence. Recently, a third transcription factor, AcuM, has been hypothesized to repress SreA and transcriptionally induce HapX via transcriptome profiling experiments [16]. Though it is unclear if the effects of AcuM on SreA and HapX are indirect or direct, AcuM null mutants have decreased siderophore production and attenuated virulence [16]. Data from these studies strongly suggest the presence of additional unidentified regulators of iron metabolism in fungi.

An appreciation for the involvement of hypoxia in fungal pathogenesis is recent and strongly supported by characterization of fungal sterol regulatory element binding protein (SREBP) null mutants that are incapable of growth in hypoxia, attenuated in fungal virulence, and more susceptible to triazole antifungal drugs [17], [18], [19], [20], [21], [22]. SREBPs are a unique family of membrane bound basic helix-loop-helix (bHLH) transcription factors that mediate a diverse array of biological processes in eukaryotic organisms [23]. In mammals, SREBPs have been observed to regulate cholesterol, lipid, and carbohydrate metabolism whereas in cholesterol auxotrophs such as *Drosophila melanogaster* and *Caenorhabditis elegans* SREBPs function to regulate fatty acid biosynthesis and development [24], [25], [26], [27], [28]. In *Schizosaccharomyces pombe and Cryptococcus neoformans*, SREBPs transcriptionally regulate genes involved in responses to low oxygen with the ergosterol biosynthesis pathway being an important downstream effector [17], [18], [29], [30]. A preliminary characterization of the *A. fumigatus* SREBP affected transcriptome adds further support to the conclusion that fungal SREBPs are key transcriptional regulators of ergosterol biosynthesis [19]. Yet, the key SREBP mediated downstream effectors in *A. fumigatus* remain to be fully elucidated. Discovering the SREBP mediated regulon in *A. fumigatus* and other human pathogenic fungi is critical for fully understanding the role of this transcriptional regulator in fungal pathogenesis.

In this study, we utilized microarray-based transcriptomics and molecular genetics to further define the role of the SREBP SrbA in *A. fumigatus*. We report that in *A. fumigatus* SrbA is an unidentified regulator of iron homeostasis. Additionally, we observe that SrbA's role in iron metabolism is intimately linked with SrbA's previously identified role in hypoxia adaptation and triazole drug susceptibility. Together, these results advance our understanding of regulation of fungal iron homeostasis and provide new evidence for understanding the role of fungal SREBPs in fungal virulence, hypoxia adaptation, and antifungal drug susceptibility.

## Results

### Hypoxia Transcriptome analysis of the *A. fumigatus* SREBP null mutant reveals downstream effectors associated with ergosterol biosynthesis and iron acquisition

**Figure 1:**
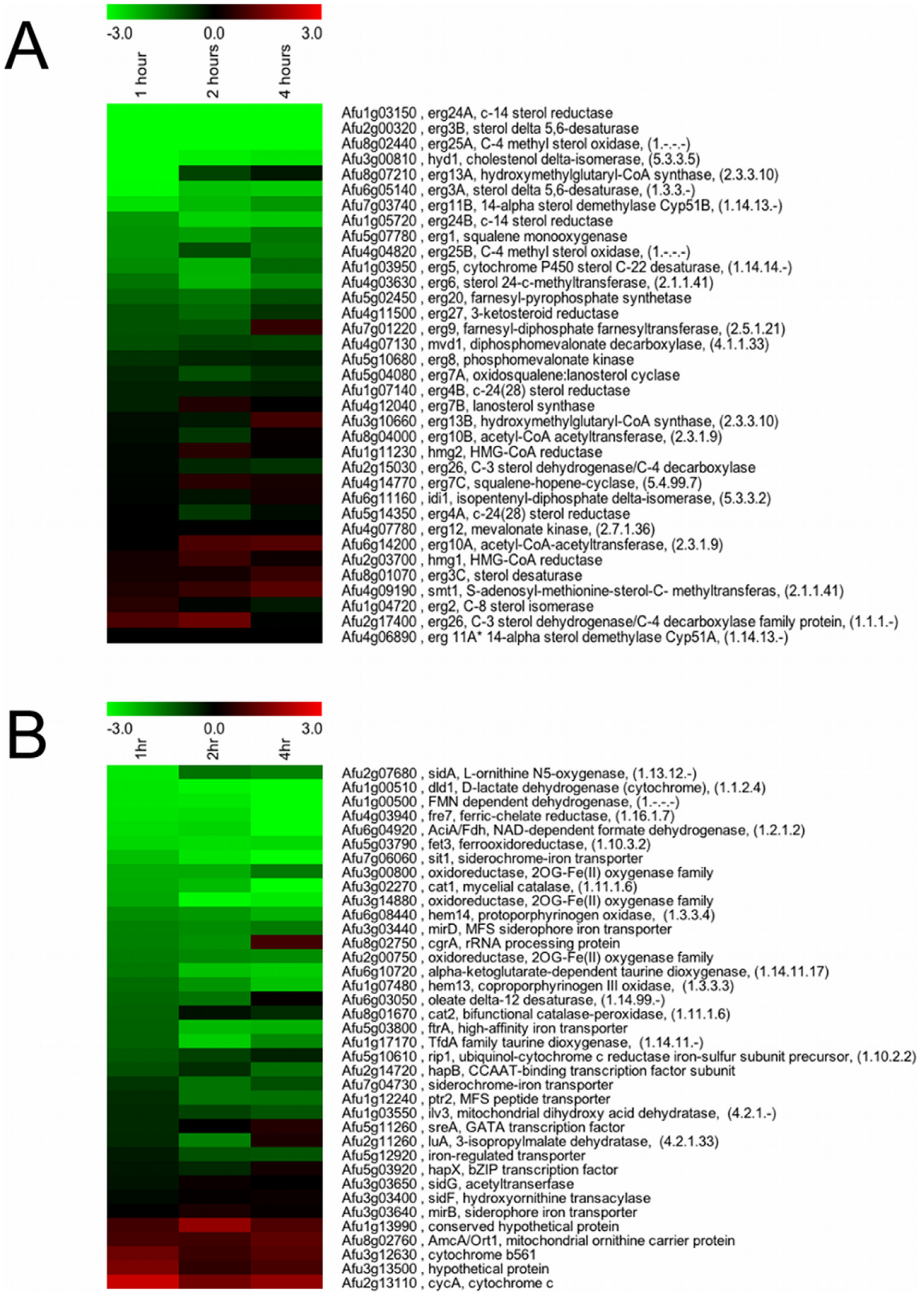
SrbA regulates genes encoding enzymes involved in ergosterol biosynthesis and iron metabolism in response to hypoxia. (A) Heat map representation of gene transcripts involved in ergosterol biosynthesis that are affected by *A. fumigatus* SrbA (B) Heat map representation of gene transcripts involved in iron metabolism that are affected by SrbA. A complete list of differentially expressed genes is available in Tables S1, S2, S3, S4. Data compare wild-type *A. fumigatus* to the Δ*srbA* strain at the indicated times after exposure to hypoxic conditions, such that wild-type transcript levels after one hour hypoxia exposure is compared to Δ*srbA* after one hour. Red indicates transcript levels are higher in Δ*srbA* (fold changes are log base 2). Three biological replicates each with dye flips were performed for each time point examined.

Previously, we reported that loss of the SREBP, SrbA, in the human fungal pathogen *A. fumigatus* resulted in loss of hypoxia growth, increased susceptibility to triazole antifungal drugs, and a significant attenuation in virulence [19]. To better understand the mechanisms of the previously observed SrbA dependent clinically relevant phenotypes, we sought to identify potential SrbA downstream effectors in *A. fumigatus*. We compared whole genome transcript level profiles of wild-type and the SREBP null mutant, Δ*srbA*, in response to hypoxia (1% O_2_, 5% CO_2_, 94% N_2_). Because Δ*srbA* cannot grow in hypoxia, a shift experiment was done whereby both strains were grown in normoxic conditions to the germling stage, then shifted to hypoxia conditioned media for defined time points. Transcriptome profiles at 1, 2, and 4 hours post exposure to hypoxia were measured with microarrays and reveal dramatic changes in the fungal transcriptome due to loss of SrbA activity (Tables S1, S2, S3, S4, S5, S6 and Figure 1). At one-hour post exposure to hypoxia, levels of mRNA from 639 genes were reduced ≥2 fold in the absence of SrbA (6.5% of the genome) (Table S1). mRNA from an additional 524 genes was increased ≥2 fold due to absence of SrbA (5.3% of the genome) (Table S2). Thus, upon initial exposure to hypoxia, approximately 12% of *A. fumigatus* genes are affected by SrbA activity. At 2 hours post-exposure to hypoxia, the number of mRNAs whose abundance decreased ≥2 fold increased to 773 (Table S3) and the number of mRNAs whose abundance increased ≥2 fold increased to 727 (Table S4). Finally, at 4 hours post-exposure to hypoxia, 602 mRNAs remained transcriptionally decreased ≥2 fold (Table S5) while 667 mRNAs remained ≥2 fold transcriptionally increased (Table S6). Manual gene ontology analysis as well as Gene set enrichment analysis (GSEA) of available GO terms suggested an SrbA dependency for ergosterol biosynthesis, iron acquisition, glycolysis, ribosome biogenesis, and amino acid biosynthesis (Figure S1, S2, S3; Tables S7, S8, S9). Taken together, these results suggest that SrbA is a major transcriptional regulator in *A. fumigatus* that may act as both a positive and negative regulator of transcription.

**Figure 2:**
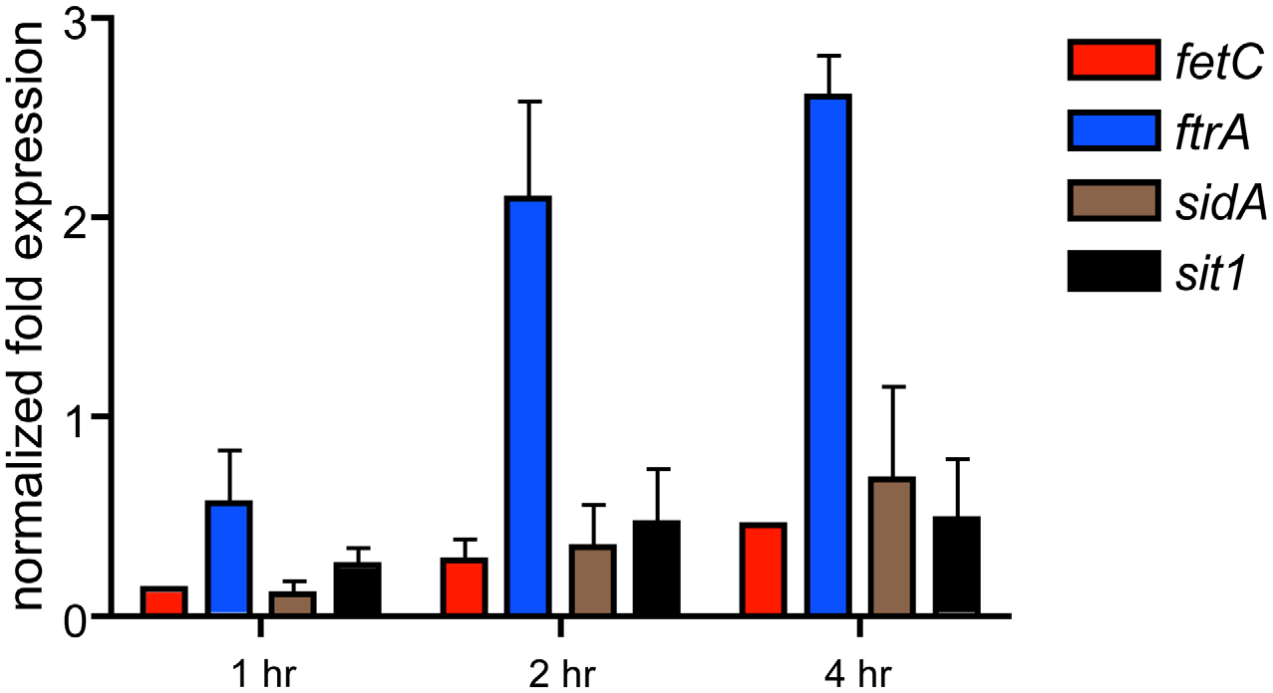
qRT-PCR confirmation of SrbA-dependent iron homeostasis gene transcript abundance in hypoxia. Transcript levels of *fetC*, *ftrA*, *sidA*, *and sit1* were examined in normoxia then after a shift to hypoxia for 1, 2, and 4 hours in wild-type CEA10 and Δ*srbA* strains. Transcript levels were normalized to β-tubulin transcript levels in each sample and data is presented relative to the wild-type transcript levels at time 0 in normoxia for each transcript using 2∧-ΔΔC_t_ method. Normalized fold expression less than 1 indicate the transcript levels are reduced in Δ*srbA* compared to wild-type.

Previous studies in *S. pombe*, *C. neoformans*, *and A. fumigatus* strongly suggested that fungal SREBPs are key regulators of ergosterol biosynthesis. Thus, not surprisingly, levels of mRNAs encoding Erg24, Erg3, and Erg25A were all at least 20 fold less abundant at one hour post-exposure to hypoxia in the absence of SrbA (Figure 1A, Table S1). The levels of mRNA from these genes remained substantially reduced at 2 and 4 hours and confirm our previously reported sterol profiles of the SrbA null mutant that demonstrated a partial block in ergosterol biosynthesis at the level of C4-demethylation [19]. In addition, and in contrast to our previously published analysis of a 24 hour time point transcriptome, levels of mRNA from several key genes encoding enzymes involved in iron homeostasis were found to be reduced at least 6 fold in the absence of SrbA (Figure 1B, Tables S1, S3, S5). The decrease in mRNA of genes associated with iron metabolism suggests that the initial response to hypoxia of *A. fumigatus* involves transcriptional induction of genes involved in iron acquisition. This result supports previous studies in mammals that demonstrate a tight link between hypoxia adaptation and iron homeostasis. In *A. fumigatus*, mRNA levels of *sidA*, an L-ornithine monooxygenase that catalyzes the first step in siderophore biosynthesis were reduced in the absence of SrbA (Figure 1B and Figure 2). Reduction in *sidA* mRNA levels would be expected to decrease both extracellular and intracellular siderophore production in *A. fumigatus*. mRNA from other genes involved in iron acquisition were also less abundant in the absence of SrbA including, the siderophore transporters *mirB* and *sit1*, the high affinity iron transporter *ftrA*, and the ferrooxidoreductase *fetC* involved in reductive iron assimilation. We further confirmed the SrbA dependency for transcription of iron associated genes in response to hypoxia utilizing qRT-PCR (Figure 2). At 1, 2, and 4 hours, exposure to hypoxia reduced transcript levels of *fetC*, *sidA*, *and sit1* in the absence of SrbA. Transcript levels of *ftrA* were SrbA dependent at 1 hour post-exposure to hypoxia, but increased at 2 and 4 hours via an unknown mechanism. Taken together, these results suggest that SrbA is a critical regulatory factor for iron homeostasis during the initial response to hypoxia.

**Figure 3:**
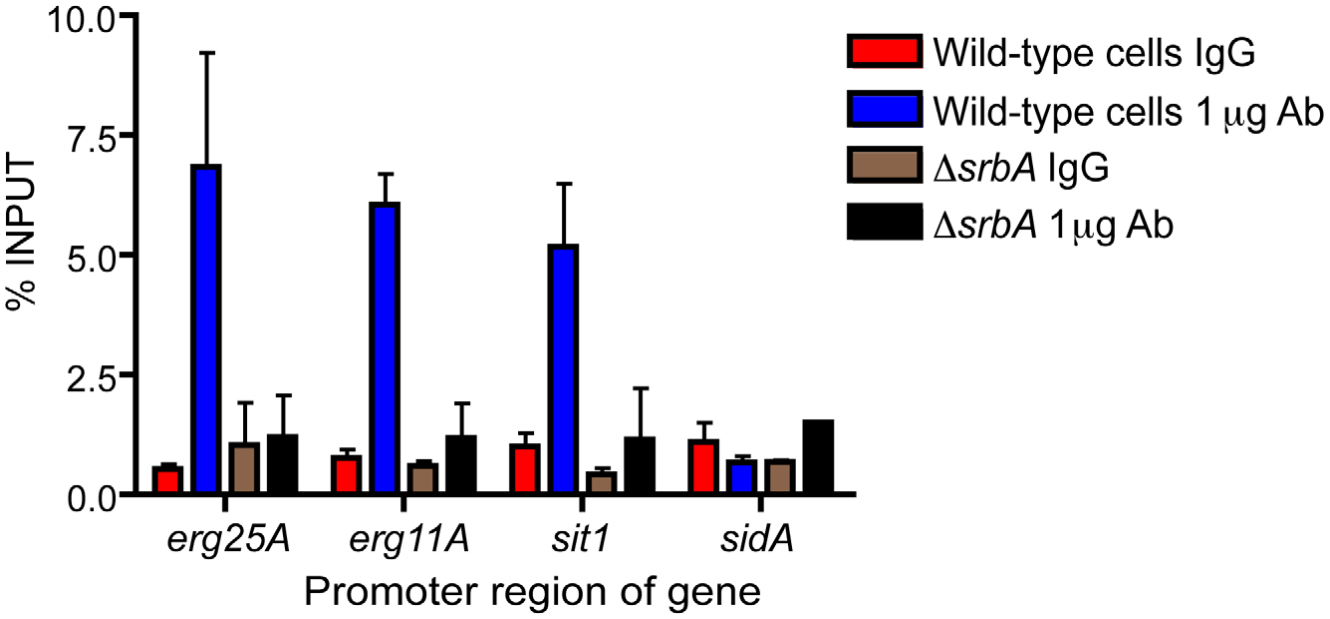
Enrichment of SrbA at the promoters of ergosterol biosynthesis and iron uptake genes. Chromatin immunoprecipitation (ChIP) qPCR was performed on DNA from wild-type and Δ*srbA* cells that were incubated in hypoxia for 4 hours. DNA was precipitated with either control IgG or 1 µg of anti-SrbA polyclonal antibody. Binding of SrbA to putative promoter regions was assessed with qPCR and data is presented as the percent enrichment of each sample to the input control. Results are the mean and standard deviation of 2 biological ChIP replicates and two qPCR technical replicates.

We next asked the question whether SrbA directly or indirectly regulated transcriptional regulation of ergosterol biosynthesis and iron acquisition. Wild-type and Δ*srbA* strains were cultivated as for the microarray and qRT-PCR experiments, and at 4 hours post-exposure to hypoxia chromatin immunoprecipitation (ChIP) was performed using IgG and polyclonal SrbA (amino acids 1–275) antibodies. Immunoprecipitated DNA was quantified for *erg25A*, *erg11A*, *sit1*, and *sidA* using primers targeted to the promoter regions of these genes. Significant enrichment for SrbA binding to the promoters of *erg11A*, *erg25A*, and *sit1*, was observed indicating that SrbA likely directly binds to the promoters of these genes (Figure 3). However, no enrichment was observed for *sidA* indicating that SrbA regulation of this important siderophore biosynthesis gene may be indirect. Taken together, these results strongly suggest that SrbA coordinately regulates ergosterol biosynthesis and iron uptake in response to hypoxia.

### SrbA-deficiency impairs submersed growth during iron starvation due to iron shortage

Because iron is a critical co-factor for enzymes involved in ergosterol biosynthesis, we explored the hypothesis that SrbA is a positive regulator of iron acquisition independent of the known fungal iron regulators SreA and HapX. In order to compare the function of SrbA with that of SreA and HapX, Δ*sreA* and Δ*hapX* mutants were generated in CEA10 and Δ*srbA* backgrounds as previously described for ATCC46645 [2], [15]. We then tested the consequences of SrbA-deficiency in the generated strains in submersed liquid cultures under iron replete and iron limiting conditions. In iron replete conditions, Δ*srbA* biomass was 54% (54.0/100.0) of the wild-type and in iron depleted conditions, Δ*srbA* biomass diminished even further to 32% (18.52/57.47) of wild-type (Table 1). Importantly, the Δ*srbA* reconstituted strain completely restored wild-type biomass in response to iron depletion indicating that the iron phenotype observed is specifically due to loss of SrbA (Table 1). Consequently, the −Fe/+Fe biomass ratio decreased from 57% (57.47/100.00) for CEA10 to 34% (18.52/54.00) for Δ*srbA* (Table 1). Thus, loss of SrbA negatively affects the ability of *A. fumigatus* to deal with low iron environments supporting the observed transcriptional profiling data.

**Table 1:** SrbA-deficiency impairs submersed biomass production in particular during iron starvation.

	CEA10	Δ*sreA*	Δ*hapX*	Δ*srbA*	Δ*srbA*Δ*sreA*	Δ*srbA*Δ*hapX*	*srbA^R^*
**−Fe**	57.47±4.59	53.95±9.8	23.69±7.66	18.52±4.28	27.28±5.81	11.63±2.08	54.82±3.98
**+Fe**	100.00±8.67	97.96±20.8	102.47±6.68	54.00±13.02	64.94±8.43	52.21±2.24	96.72±9.06
**ratio** **−/+Fe**	0.57	0.55	0.23	0.34	0.42	0.22	0.57

Biomass production (dry mass) of 10^8^ conidia in 200 ml liquid AMM was scored during iron starvation (−Fe) and iron sufficiency (+Fe, 30 µM) after 24 h incubation at 37°C at 200 rpm and normalized to that of the CEA10 grown under iron sufficiency. The given values are the mean ± STD of six biological replicates.

As expected, SreA-deficiency did not affect liquid biomass production during either iron-replete or iron-depleted conditions. Intriguingly, inactivation of SreA in the Δ*srbA* strain increased fungal biomass by 47% in iron-depleted conditions and 20% in iron-replete conditions compared to the Δ*srbA* strain itself. Consequently, this increased the −Fe/+Fe biomass ratio to 42% (Table 1). As found previously for *A. fumigatus* strain ATCC46445 [2], HapX-deficiency decreased liquid biomass production during iron starvation in *A. fumigatus* strain CEA10 by about 60% but had no significant effect during iron sufficiency because *hapX* is mainly expressed during iron starvation [2]. Compared to Δ*srbA*, additional inactivation of HapX in Δ*srbA* decreased the biomass by 37% in iron depleted conditions but had no effect in iron-replete conditions (Table 1). Taken together, these data strongly support the hypothesis that SrbA is required for adaptation to iron starvation independent of the known fungal iron metabolism regulators SreA and HapX.

### SrbA is required for full activation of extra- and intracellular siderophore production

**Figure 4:**
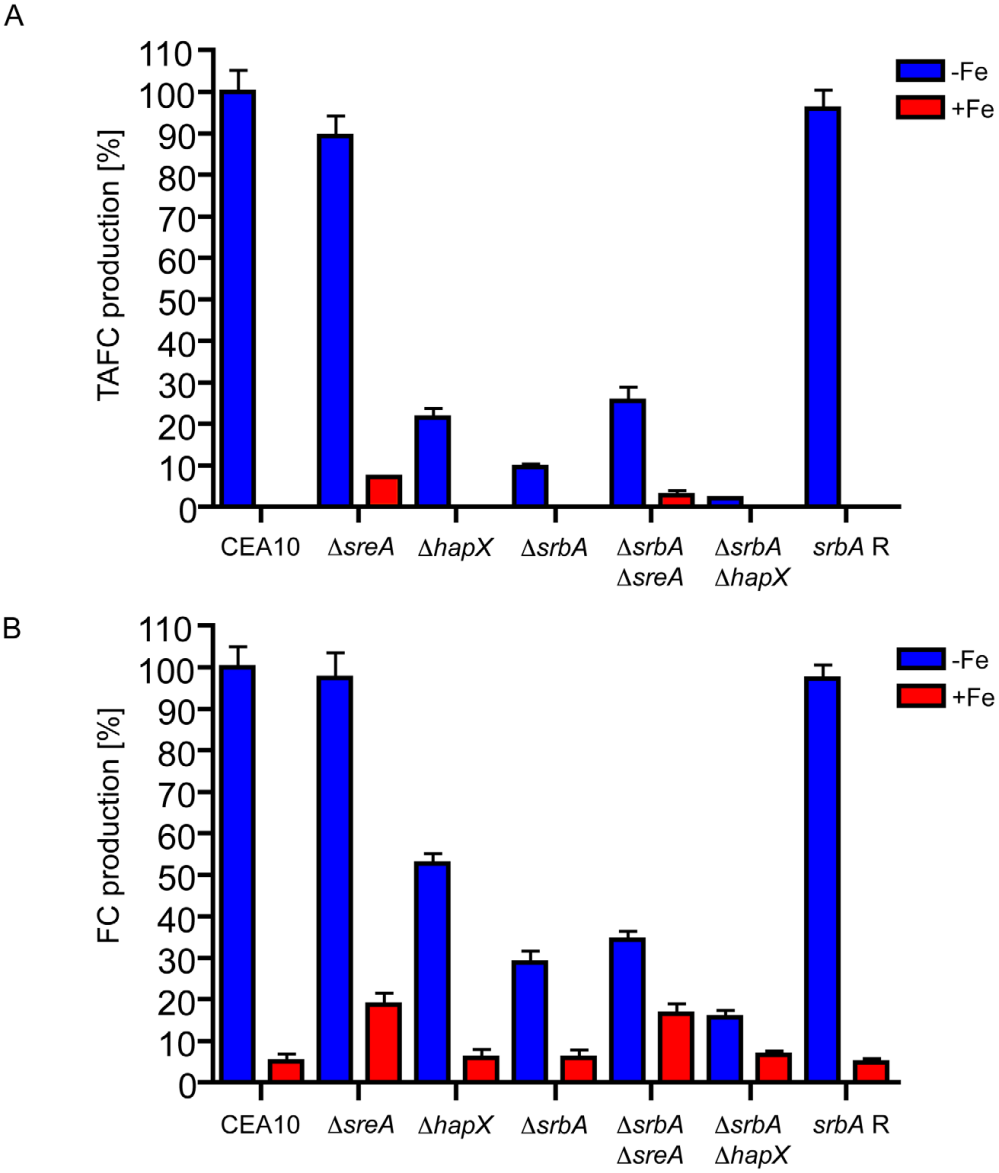
SrbA activates extracellular and intracellular siderophore production independent of SreA and HapX. (A) Extracellular and (B) intracellular. Quantification of extracellular TAFC and intracellular FC and after growth for 24 hours at 37°C during iron-replete (+Fe) and depleted (−Fe) conditions was normalized to the biomass of the respective strain and furthermore to that of the CEA10 during iron starvation. The given values are the mean ± SD of six biological replicates. Under iron-replete conditions the measured FC was iron-free (desferri-FC), while under iron-replete conditions the FC was iron loaded (ferri-FC).

We next explored the mechanism behind the detrimental effects of iron starvation on Δ*srbA*. Our transcriptome profiling experiments implied a potential role for SrbA regulation of siderophore biosynthesis and uptake. As shown previously for *A. fumigatus* strain ATCC46445 [4], [5], *A. fumigatus* strain CEA10 produces extracellular TAFC (triacetylfusarinine C) exclusively during iron starvation. Intriguingly, SrbA-deficiency decreased TAFC production by 90% compared to wild-type CEA10 in iron starvation conditions (Figure 4A). Similar to previous findings with *A. fumigatus* strain ATCC46445 [2], [15], HapX-deficiency in CEA10 decreased TAFC production during iron starvation by 79% during iron starvation while SreA-deficiency in CEA10 caused a 7% derepression of TAFC production during iron sufficiency (Figure 4A). Compared to Δ*srbA*, additional deletion of *hapX* in Δ*srbA* decreased TAFC production by 79% during iron depleted conditions (Figure 4A). In contrast, additional deletion of *sreA* in Δ*srbA* increased TAFC production compared to Δ*srbA* by 163% during iron starvation and derepressed TAFC production to 40% of the Δ*sreA* level during iron sufficiency (Figure 4A).

As shown previously for *A. fumigatus* strain ATCC46445 [4], [5], *A. fumigatus* strain CEA10 accumulates intracellular FC (ferricrocin) in the ferri-form during iron sufficiency and about 20 fold higher amounts in the desferri-form during iron starvation (Figure 4B). SrbA-deficiency had no effect of the FC during iron sufficiency. However, SrbA-deficiency decreased the FC content by 71% compared to CEA10 during iron starvation (Figure 4B). Similar to previous findings with *A. fumigatus* strain ATCC46445 [2], [15], HapX-deficiency in CEA10 decreased the FC content during iron starvation by 47% during iron starvation while SreA-deficiency in CEA10 increased the FC content during iron sufficiency 3.7 fold (Figure 4B). Compared to Δ*srbA*, the FC content in Δ*srbA*Δ*hapX* is decreased by 46% during iron starvation (Figure 4B). In contrast, additional deletion of SreA in Δ*srbA* increased the FC content compared to Δ*srbA* by 19% during iron starvation and 181% during iron sufficiency (Figure 4B).

Together, these data indicate that SrbA activates production of extra-and intracellular siderophores independent of SreA and HapX. Moreover, this biochemical data supports the observed transcriptome profile of Δ*srbA* that strongly suggests a critical role for SrbA in regulation of siderophore biosynthesis and uptake. However, the mechanism by which SrbA regulates siderophore production remains to be elucidated. As production of extra- and intracellular siderophores plays a crucial role in adaptation to iron starvation [4], [5], these data explain, at least in part, the observed growth and morphological defects of Δ*srbA* during iron starvation.

### srbA mRNA abundance is regulated by iron availability, and SrbA positively affects iron acquisition and biosynthesis of heme and ergosterol

**Figure 5:**
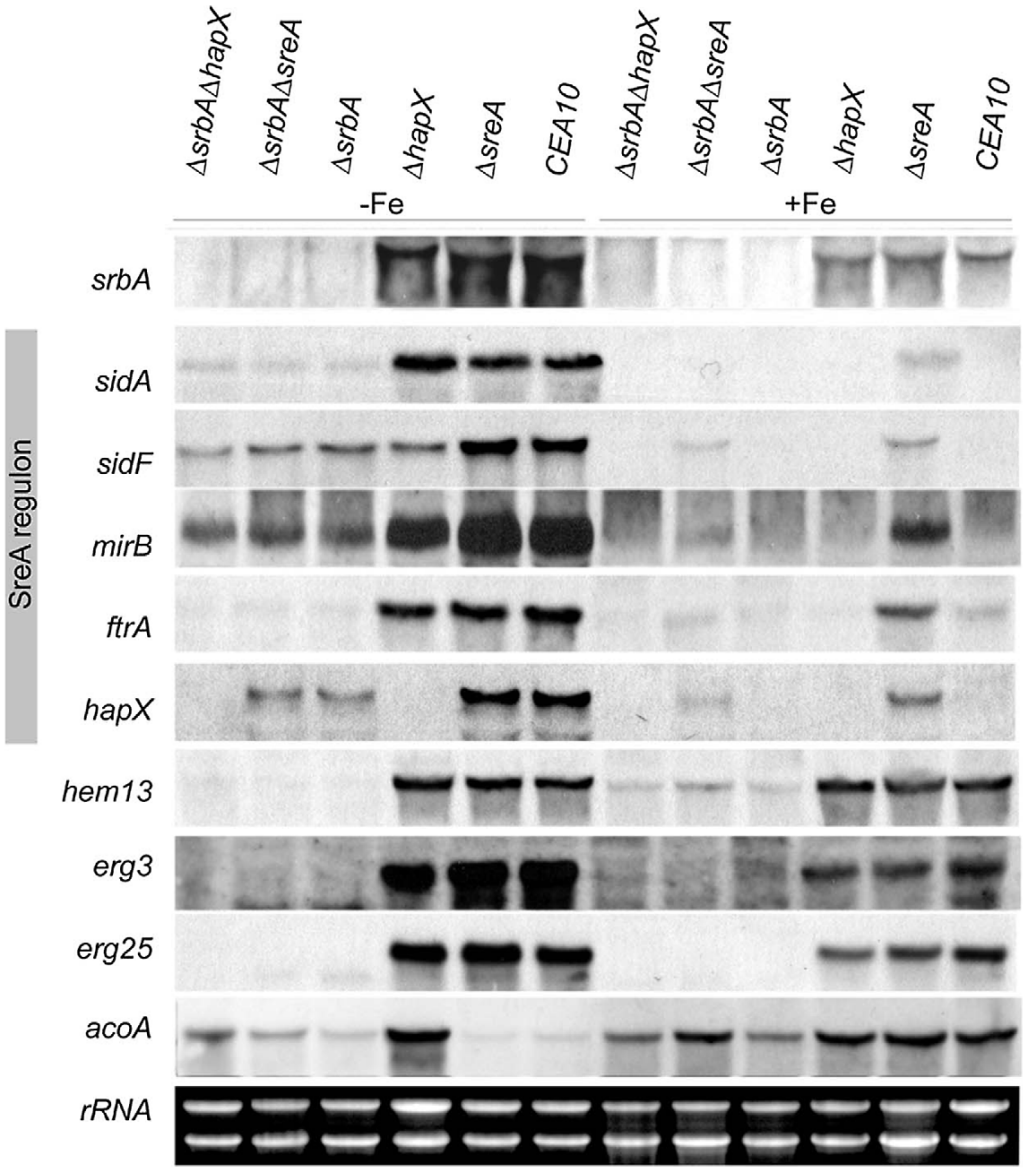
*srbA* expression is transcriptionally upregulated by iron starvation and SrbA-deficiency downregulates the SreA regulon independent of SreA and HapX, as well as the ergosterol biosynthetic *erg3* and *erg25* and the heme-biosynthetic *hem13*. For Northern analysis, total RNA was isolated from *A. fumigatus* strains grown for 24 h in liquid cultures at 37°C at 200 rpm during iron-replete (+Fe) and depleted (−Fe) conditions. Ethidium bromide-stained rRNA is shown as control for loading and quality of RNA.

In further support of SrbA's role as a positive regulator of iron homeostasis, previous genome-wide transcriptome profiling experiments indicated that *srbA* transcript levels are reduced within 30–60 minutes during a shift from iron starvation to iron sufficiency independent of SreA and HapX [2], [15]. Thus, we next confirmed that *srbA* transcript levels are substantially higher during iron starvation compared to iron sufficiency, and that *srbA* transcript levels are not influenced by inactivation of SreA or HapX (Figure 5). These results further support the hypothesis that SrbA transcript levels increase under low iron conditions and that this increase is independent of the known transcriptional regulators of iron homeostasis SreA and HapX.

Consistent with the transcriptome profile of Δ*srbA* upon early exposure to hypoxia and biochemical analysis of siderophore production in the absence of SrbA, inactivation of SrbA reduced mRNA levels of genes involved in siderophore metabolism (siderophore biosynthetic *sidA*, TAFC-biosynthetic *sidF*, and siderophore importer-encoding *mirB*), reductive iron assimilation (*ftrA*), and iron transcriptional regulation (*hapX*) in iron limited conditions (Figure 5). All of these genes belong to the SreA regulon [15]. Moreover, SrbA deletion decreased the degree of derepression of these genes in Δ*sreA* during iron sufficiency (compare Δ*sreA* and Δ*srbA*Δ*sreA*). The reduction of *hapX* mRNA levels in the absence of SrbA in iron limiting conditions suggests a previous unreported link between SrbA and HapX in iron limiting conditions. Together, these data support a role for SrbA in adaptation to iron starvation and demonstrate that SrbA impacts siderophore biosynthesis at the transcriptional level both in response to hypoxia and iron starvation through an unknown mechanism.

Importantly, mRNA levels of genes involved in ergosterol biosynthesis were also found to be more abundant under iron starvation conditions (Figure 5). Similar to the hypoxia mRNA transcriptome profiling data, this increase in mRNA levels is SrbA dependent (Figure 5). In further support of SrbA's role in regulating iron metabolism, mRNA levels of the iron center-ergosterol biosynthetic enzymes Erg3 (C-5 sterol desaturase) and Erg25 (C-4 methyl sterol oxidase) were independent of SreA and HapX (Figure 5). As mRNA levels from both *erg3* and *erg25* are also reduced in Δ*srbA* in response to hypoxia, these data further support the SrbA mediated link between ergosterol biosynthesis and iron metabolism in response to hypoxia in *A. fumigatus*. Also of interest, SrbA inactivation decreased mRNA levels of the heme biosynthetic gene *hem13* (encoding coproporphyrinogen III oxidase) independent of SreA and HapX in iron limited and hypoxia conditions.

Importantly, mRNA levels of *acoA*, which encodes the iron-sulfur cluster-containing aconitase and whose expression is subject to HapX-mediated repression during iron starvation are decreased during iron-replete conditions in Δ*srbA and* Δ*srbA*Δ*hapX* but not in Δ*srbA*Δ*sreA* [2]. These data indicate that SrbA-deficiency decreases cellular iron supply during iron-replete conditions due to reduction of iron uptake. Importantly, this defect can be partially suppressed by derepression of iron uptake via SreA-deficiency (Δ*srbA*Δ*sreA*).

### Increased iron availability and/or inactivation of SreA increases resistance of ΔsrbA to fluconazole and partially restores growth in hypoxia

**Figure 6:**
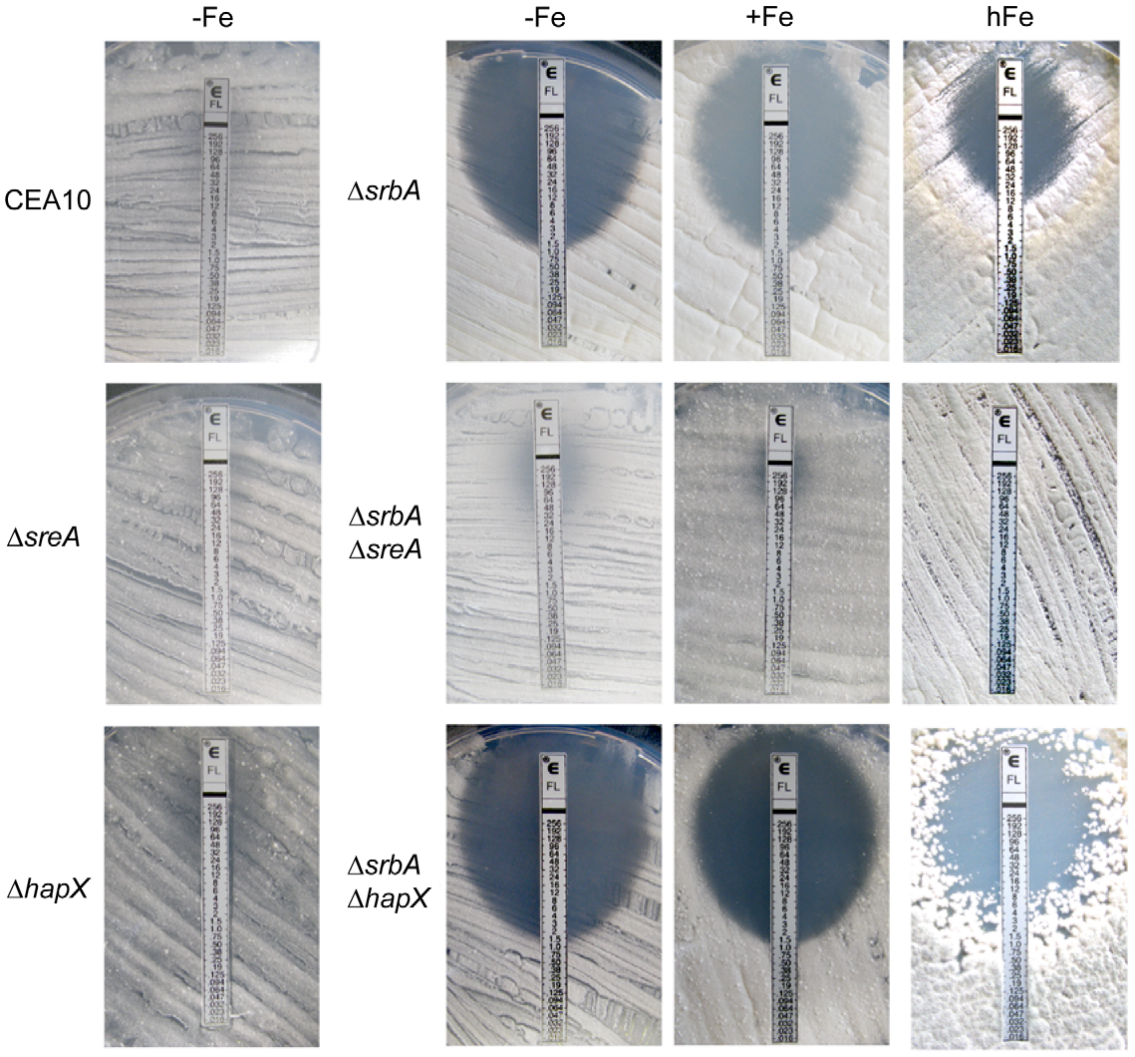
Increased iron availability and/or inactivation of SreA increase resistance of Δ*srbA* to fluconazole. E-test strips (AB Biodisk, bioMérieux) impregnated with a gradient of fluconazole were placed onto a MM agar plates representing different iron availability (−Fe; +Fe, 30 µM; hFe,10 mM iron) and containing a lawn of conidia. Growth inhibition was measured after 48 h at 37°C by direct observation.

We next explored the hypothesis that the partial suppression of decreased cellular iron supply in Δ*srbA*Δ*sreA* would rescue the clinically relevant phenotypes of Δ*srbA*: increased fluconazole susceptibility, inability to grow in hypoxia, and ability to cause invasive pulmonary aspergillosis. E-test mediated fluconazole susceptibility testing confirmed our previously published results that the inherent fluconazole resistance of *A. fumigatus* CEA10 depends on SrbA activity (Figure 6). Fluconazole susceptibility in Δ*srbA* was consistent in both iron depleted and iron replete conditions. Intriguingly, high iron conditions were able to increase the resistance of Δ*srbA* against fluconazole (high iron MIC = 12 µg/ml compared to an MIC of 1 µg/ml during iron replete or iron starvation). Additional deletion of *sreA*, but not *hapX*, in Δ*srbA* partially rescued fluconazole resistance, and this effect, as expected, was potentiated under high iron conditions. Importantly, deletion of either *sreA* or *hapX* alone did not affect fluconazole susceptibility. Taken together, these results suggest that the increase in *A. fumigatus* fluconazole susceptibility in the absence of SrbA is partially due to loss of iron homeostasis.

**Figure 7:**
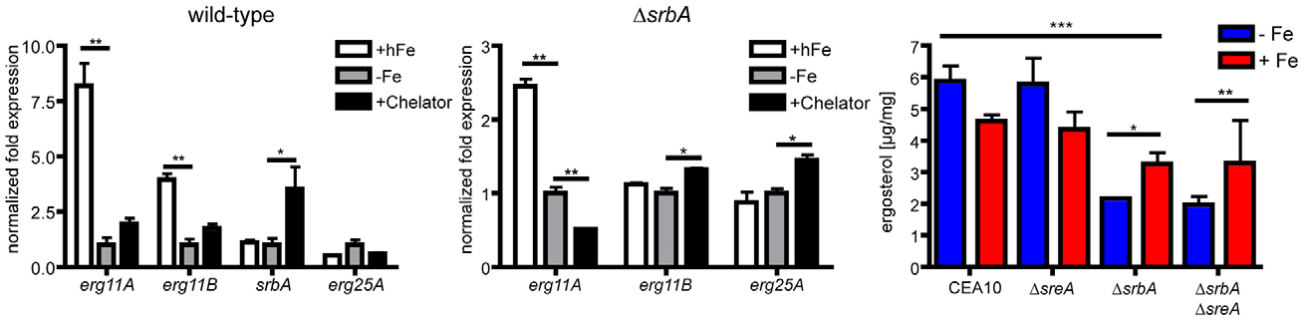
Increased iron levels increase *erg11A* transcript and total ergosterol levels in the absence of SrbA. qRT-PCR analysis of *erg11A*, *erg11B*, *srbA*, *and erg25A* transcript levels were measured in the wild-type CEA10 (A) and Δ*srbA* strains (B). Transcript levels were normalized to *tefA* transcript levels in each sample and data was normalized to the −Fe sample in both strains examined. Chelator = 100 µM of the iron chelator 2,2-dipyridyl was added to the culture medium to completely remove free iron. Data represents the mean and standard deviation of three biological and two PCR technical replicates. (C) Total ergosterol content of respective strains in response to iron depleted and high iron conditions. Data represent the mean and standard deviation of 2 biological replicates. *,**, *** = p<0.05, two-tailed paired t-Test. *** refers to statistical comparisons between CEA10 in both Fe+ and Fe− to Δ*srbA* in both Fe+ and Fe−.

The direct binding of SrbA to the promoter of *erg11A* (also called *cyp51A* in *A. fumigatus*) led us to explore the potential mechanism behind this result. We thus examined transcript levels of *erg11A*, *erg11B (cyp51B)*, *erg25A*, *and srbA* in response to varying levels of iron in wild-type and Δ*srbA*. Addition of high iron to either wild-type or Δ*srbA* significantly increased *erg11A* transcript levels (Figure 7A and 7B). As expected from the ChIP experiment, this effect on *erg11A* transcript was SrbA dependent (Figure 7A and 7B). Of note, *erg11A* is not contained on the microarray and thus *erg11A* transcript levels were not previously observed to be SrbA dependent. Consistent with the previous Northern blot experiments, loss of iron stimulated an increase in *srbA* transcript levels (Figure 7A). However, the effect of iron on *erg25A* transcripts was minimal, though as observed with the microarray data, *erg25A* transcript levels are SrbA dependent (Figure 7A and 7B). Next, we examined total ergosterol levels in wild-type, Δ*srbA*, and Δ*srbA*Δ*sreA* strains (Figure 7C). Addition of high iron was able to increase total ergosterol levels in the Δ*srbA* and Δ*srbA*Δ*sreA* backgrounds consistent with the increase in *erg11A* transcript levels. No difference in ergosterol levels was observed between the wild-type and Δ*sreA* strains (Figure 7C). These data, however, do not rule out the potential for increased enzyme efficiency in the presence of more available iron, or a restoration of membrane fluidity due to increases in ergosterol level that may affect triazole drug uptake. However, these data suggest that loss of iron homeostasis due to absence of SrbA affects ergosterol biosynthesis and triazole drug interactions in *A. fumigatus*.

**Figure 8:**
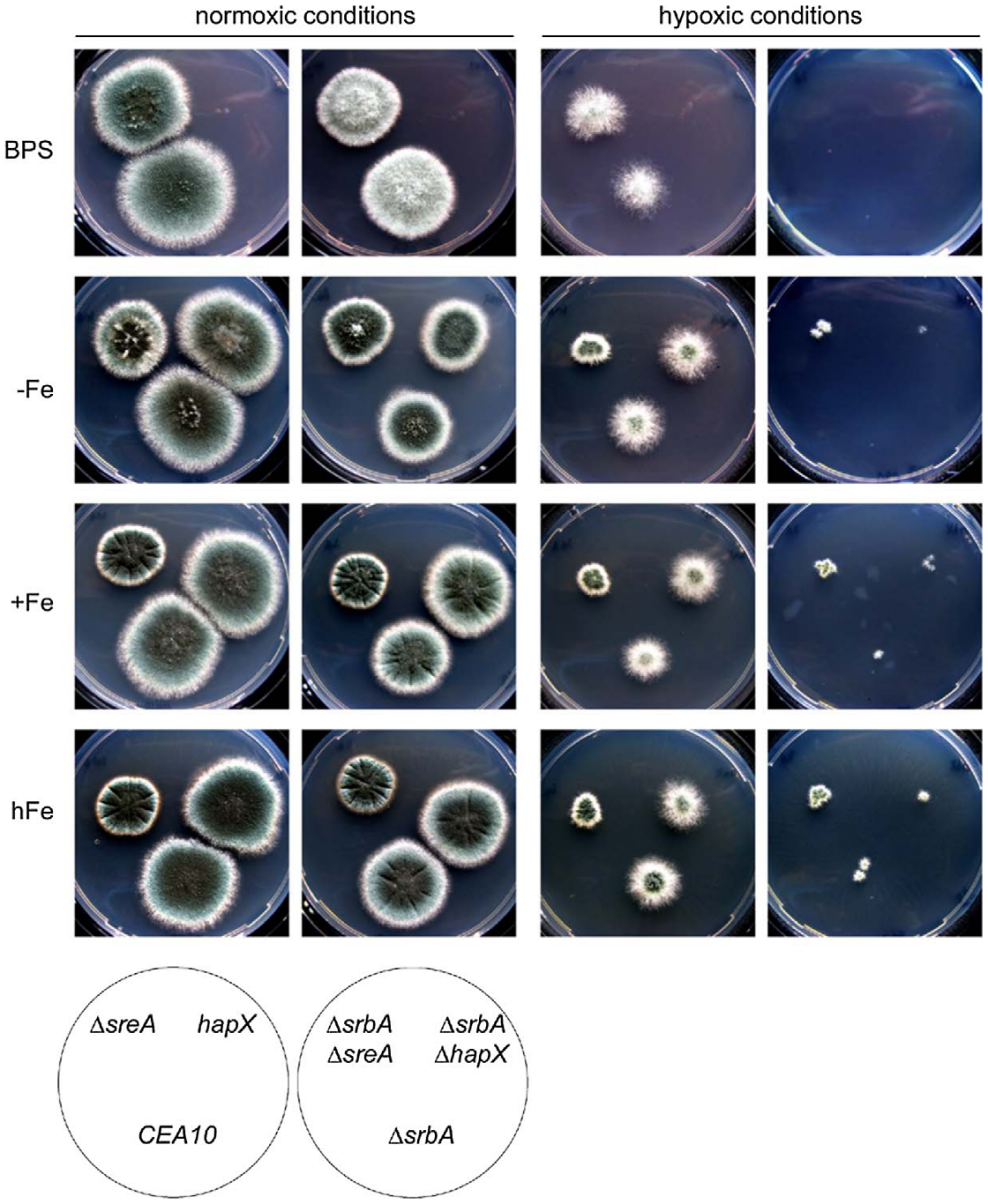
Increased iron availability and/or inactivation of SreA improve growth of Δ*srbA* during hypoxia. For plate growth assays of CEA10, Δ*sreA*, Δ*hapX*, Δ*srbA*, Δ*srbA*Δ*sreA*, and Δ*srbA*Δ*hapX* under normoxic and hypoxic conditions, 100 conidia of each strain were point-inoculated on AMM agar plates containing different iron concentrations (−Fe; +Fe, 30 µM; hFe,1.5 mM) or the iron chelator BPS (−Fe, 100 µM BPS) and incubated at 37°C for 96 h during hypoxic conditions or normoxic conditions.

As high iron levels or deletion of SreA were able to partially rescue the fluconazole susceptibility and decrease in ergosterol content in Δ*srbA*, we hypothesized that these effects may also rescue the hypoxia growth defect of Δ*srbA*. In further support of a link between hypoxia adaptation and iron homeostasis in *A. fumigatus*, supplementation of media with high iron concentrations plus inactivation of SreA partially rescues growth of Δ*srbA* in hypoxia (Figure 8). This result can be explained by derepression of iron uptake due to SreA inactivation, which works best in the presence of high iron concentrations. Thus, in the absence of SreA, iron uptake is increased in Δ*srbA*. HapX-deficiency had no effect on hypoxic growth and not surprisingly, is not transcriptionally induced in response to hypoxia. These data indicate that the hypoxic growth defect of Δ*srbA* is at least partially explained by a defect in iron accumulation. In further support of this conclusion, susceptibility to cobalt chloride in the absence of SrbA is also rescued by further inactivation of SreA (Figure S4). Thus, the ability of either high iron or loss of SreA activity to partially rescue the fluconazole and hypoxia phenotypes of Δ*srbA* strongly suggests that Δ*srbA* cells are iron deficient. These results further support an important link between iron homeostasis and ergosterol biosynthesis as mediated by SrbA.

**Figure 9:**
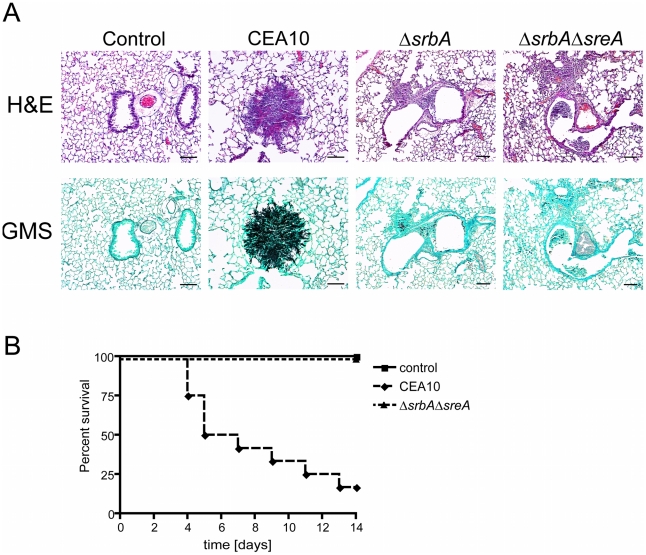
Inactivation of SreA in Δ*srbA* does not restore fungal virulence. (A) Lung histopathology of CD1 mice infected with respective *A. fumigatus* strains on day +4 after infection. Substantial fungal growth and tissue necrosis are observed in lungs of mice infected with wild-type CEA10. However, little to no fungal growth is observed in lungs of mice infected with either Δ*srbA*, *or* Δ*srbA*Δ*sreA*. (B) Kaplan-Meier survival analysis of respective *A. fumigatus* strains in chemotherapeutic model of invasive pulmonary aspergillosis. As with previously published results with strain Δ*srbA*, strain Δ*srbA*Δ*sreA* has a significant reduction in virulence compared to wild-type CEA10 (P<0.0001, Log-Rank Test for comparison between Δ*srbA*Δ*sreA* and wild-type CEA10,).

As the avirulence phenotype of Δ*srbA* is hypothesized to at least partially be the result of its inability to grow in hypoxia, we next tested the ability of Δ*srbA*Δ*sreA* to cause disease in a chemotherapeutic murine model of invasive pulmonary aspergillosis. We have previously shown that Δ*srbA* is fully avirulent in this murine model of IPA; however, inactivation of SreA in Δ*srbA* was not able to rescue virulence in the absence of SrbA (Figure 9A). Histopathological examinations of wild-type, Δ*srbA*, and Δ*srbA*Δ*sreA* strains revealed significant fungal growth and tissue necrosis in mice infected with CEA10 (Figure 9B). However, as previously reported, a significant reduction in Δ*srbA* growth is observed *in vivo* and further inactivation of SreA did not visibly change the observed histopathology. As iron availability is extremely limited *in vivo*, and previous results demonstrating that *A. fumigatus* strains defective in siderophore biosynthesis have attenuated virulence, this result is likely not surprising and does not rule out a role for SrbA mediated iron homeostasis in the avirulence phenotype of Δ*srbA*. Additional experiments examining the impact of loss of iron homeostasis in Δ*srbA* on *A. fumigatus* virulence are ongoing.

### SrbA-deficiency alters the free amino acid pool composition in *A. fumigatus* during iron sufficiency and starvation

**Figure 10:**
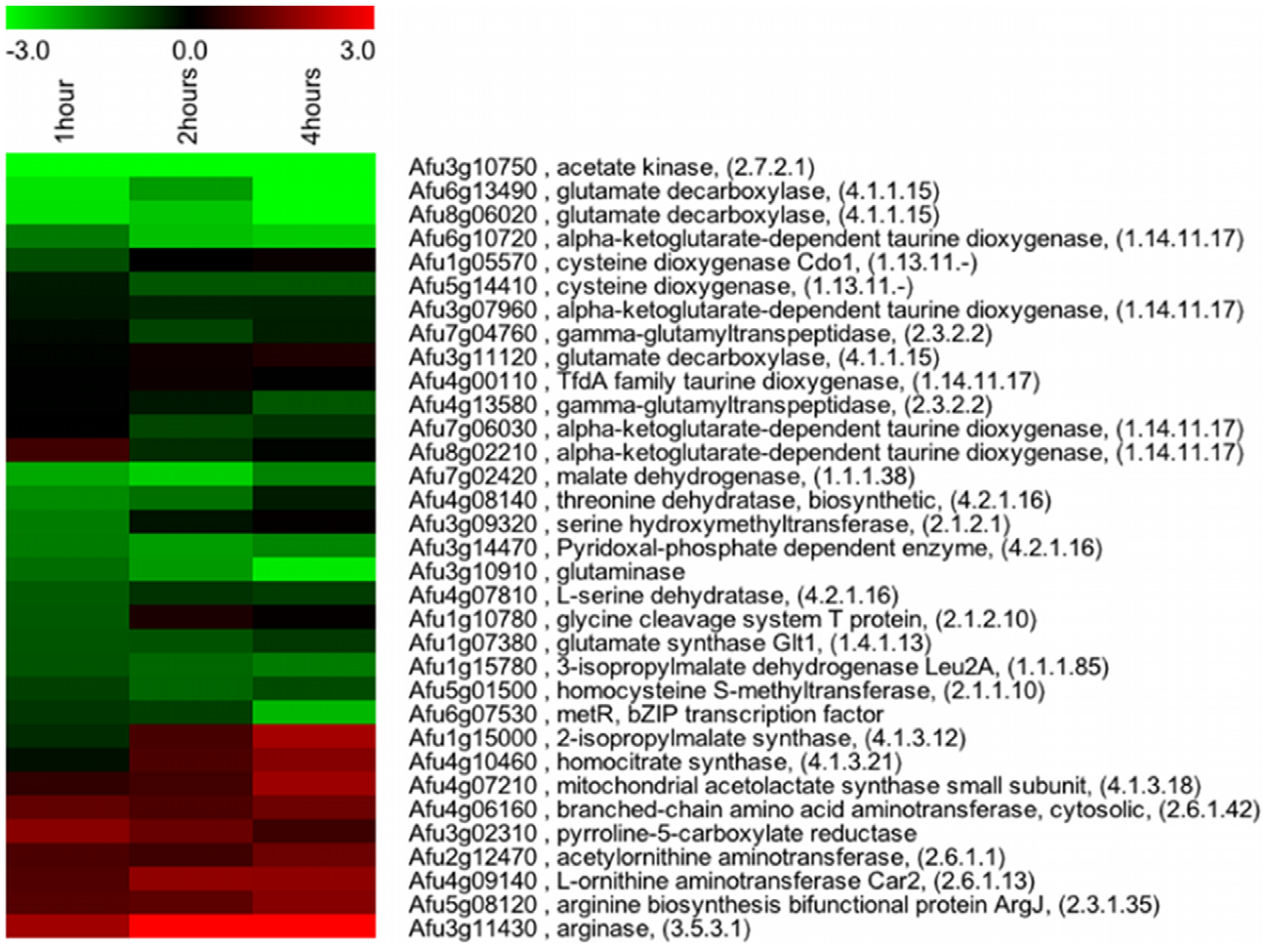
Genes involved in amino acid biosynthetic processes are transcriptionally affected by loss of SrbA in hypoxia. Heat map representation of genes involved in amino acid biosynthesis that are regulated by *A. fumigatus* SrbA. A detailed list of genes and fold changes is available in Tables S1, S2, S3, S4. Data compare wild-type *A. fumigatus* to the Δ*srbA* strain at the indicated times after exposure to hypoxic conditions, such that wild-type transcript levels after one hour hypoxia exposure is compared to Δ*srbA* after one hour. Red indicates transcript levels are higher in Δ*srbA* (fold changes are log base 2). Three biological replicates each with dye flips were performed for each time point examined.

Iron starvation has previously been observed to cause a significant remodeling of the amino acid pool [2]. HapX, which is activated by iron-starvation, affects the amino acid composition during iron starvation but not during iron sufficiency and is crucial for coordination of the production of siderophores and their precursor ornithine. Given SrbA's role in mediating responses to low iron conditions and reduction of siderophore biosynthesis in the absence of SrbA, we next tested the hypothesis that loss of SrbA would also alter the amino acid pool of *A. fumigatus*. In support of this hypothesis, the transcriptome profile data suggest significant changes in the mRNA levels of genes involved in amino acid biosynthesis in the absence of SrbA upon exposure to hypoxia (Figure 10). In contrast to HapX-deficiency, SrbA-deficiency dramatically changed the composition of the amino acid pool during both iron-replete and depleted conditions (Table 2). Similar to HapX-deficiency, SrbA-deficiency decreased the cellular ornithine pool during iron starvation, which indicates together with the decrease in siderophore biosynthesis, that SrbA also plays a role in supply of the siderophore precursor ornithine. Thus, loss of siderophore biosynthesis in Δ*srbA* may be due to regulation of critical precursor levels.

**Table 2:** Total free amino acid pool is altered in absence of SrbA.

aa	CEA10			Δ*srbA*			Δ*srbA*/CEA10	
	+Fe	−Fe	−/+ Fe	+Fe	−Fe	−/+ Fe	+Fe	−Fe
Ala	37.64±0.32	9.41±2.74	0.25**^d^**	18.14±0.73	5.73±1.64	0.32**^d^**	0.48**^c^**	0.61**^c^**
Arg	1.71±0.24	16.32±0.52	9.57**^b^**	2.77±0.74	12.59±1.41	4.55**^b^**	1.62**^a^**	0.77
Asn	1.19±0.18	3.62±0.09	3.04**^b^**	4.88±0.41	6.11±0.09	1.25	4.10**^b^**	1.69**^a^**
Asp	4.31±0.32	3.15±1.07	0.73	7.52±1.01	5.37±1.01	0.71	1.75**^a^**	1.71**^a^**
Gln	6.48±0.44	39.29±4.43	6.07**^b^**	29.96±3.45	45.88±1.25	1.53**^a^**	4.63**^b^**	1.17
Glu	40.89±0.82	9.1±2.18	0.22**^d^**	29.01±2.29	12.07±2.61	0.42**^c^**	0.71	1.33
Gly	1.74±0.03	1.83±0.25	1.05	1.1±0.03	0.87±0.14	0.80	0.63**^c^**	0.48**^c^**
His	0.28±0.03	2.29±0.38	8.06**^b^**	0.49±0.43	2.15±0.64	4.39**^b^**	1.73**^a^**	0.94
Ile	0.41±0.03	0.52±0.07	1.26	0.49±0.01	0.33±0.08	0.66**^c^**	1.20	0.63**^c^**
Leu	0.52±0.03	0.95±0.22	1.84**^a^**	0.71±0.01	0.53±0.20	0.74	1.38	0.56**^c^**
Lys	1.86±0.14	5.52±0.45	2.96**^a^**	1.44±0.03	4.24±0.36	2.94**^a^**	0.77	0.77
Met	0.07±0.00	0.25±0.01	3.45**^b^**	0.18±0.03	0.21±0.03	1.20	2.41**^a^**	0.84
Orn	0.49±0.02	4.96±0.06	10.03**^b^**	0.36±0.05	1.65±0.27	4.63**^b^**	0.72	0.33**^d^**
Phe	0.16±0.21	0.29±0.12	1.86**^a^**	0.44±0.01	0.33±0.15	0.74	2.78**^a^**	1.11
Ser	2.25±0.17	2.48±0.04	1.10	2.52±0.15	1.95±0.03	0.77	1.12	0.79

Individual amino acid pools (aa) are given in % of the total free amino acids ± STD. aa upregulated >1.5 and >3 fold in CEA10 or Δ*srbA* during iron starvation compared to iron sufficiency are marked in ^a^ and ^b^, respectively; aa pools down-regulated >1.5 and >3 fold are marked in ^c^ and ^d^ respectively. aa pools upregulated >1.5 and >3 fold in Δ*srbA* compared to CEA10 in the same condition are marked in ^a^ and ^b^, respectively; aa pools down-regulated >1.5 and >3 fold are marked in ^c^ and ^d^, respectively.

## Discussion

Understanding the *in vivo* microenvironment conditions encountered by human pathogenic fungi is a promising line of inquiry for identifying novel therapeutic options for these frequently lethal infections. The importance of iron availability in host pathogen interactions is well established, and its role in invasive pulmonary aspergillosis is no exception. Previous studies have clearly demonstrated a critical role for iron acquisition mechanisms in fungal pathogenesis for *A. fumigatus* and other human pathogenic fungi [4], [5], [7], [8], [31], [32], [33], [34], [35], [36], [37], [38], [39], [40]. More recently, it has been hypothesized that adaptation to low oxygen microenvironments during fungal infection may also be a critical virulence attribute of human pathogenic fungi [17], [18], [19], [20], [22]. Support for this hypothesis partially stems from studies with fungal SREBP null mutants in *C. neoformans and A. fumigatus* that are incapable of growth in hypoxia and unable to cause lethal disease in murine models of fungal infections [17], [18], [19], [41], [42]. However, as SREBPs are transcription factors that regulate a significant number of genes in fungi, it is unclear if the hypoxia growth phenotype of fungal SREBP null mutants is the primary factor for loss of virulence in these mutants. Moreover, fungal SREBP mutants display increased susceptibility to the triazole class of antifungal drugs.

Thus, several key questions remain regarding the role of SrbA in fungal pathogenesis. Important questions include what is the mechanism behind the inability of SREBP null mutants to grow in hypoxia? Does this directly correlate with the avirulence of fungal mutants that lack SREBPs? And what is the mechanism behind the increased susceptibility to triazole drugs in the absence of SREBP? To begin to answer these potentially clinically relevant questions, we utilized whole-genome transcriptome analysis of *A. fumigatus* Δ*srbA* exposed to hypoxia to identify SrbA downstream effectors. Here, we report that the *A. fumigatus* SREBP is a key positive regulator of iron homeostasis, particularly with regard to iron acquisition, that is essential for adaptation to hypoxia and low iron microenvironments. Although previous transcriptome profiling experiments with the *C. neoformans* SREBP null mutant also suggest a potential role for fungal SREBPs in iron acquisition [7], [17], here we definitively show that SREBP is required for adaptation to low iron conditions in *A. fumigatus*. We further observe that Δ*srbA* cells are likely iron deficient and this partially explains the hypoxia growth and triazole susceptibility phenotypes of Δ*srbA*. Importantly, SrbA's effect on iron homeostasis appears to be primarily independent of the well-studied iron transcriptional regulatory factors HapX and SreA.

SreA-deficiency in *A. fumigatus* and *A. nidulans* has been observed to partially derepress siderophore production and expression of respective genes involved in iron acquisition in iron replete conditions [15], [43]. Importantly, this result strongly suggested the existence of additional regulatory mechanisms involved in iron homeostasis in *A. fumigatus*. Next, the transcription factor HapX was demonstrated to be required not only for repression of iron-consuming pathways but also for activation of siderophore biosynthesis and uptake during iron starvation in *A. nidulans* and *A. fumigatus* [2], [44]. SreA and HapX are interconnected by a negative transcriptional feed back loop and simultaneous inactivation has been shown to be synthetically lethal in *A. nidulans* and *A. fumigatus* [2], [44]. Recently, the transcription factor AcuM, that is required for gluconeogenesis, was found to also activate siderophore biosynthesis most likely via repression of SreA in *A. fumigatus* but not *A. nidulans* [16]. Here we present data that strongly suggest that SrbA is another critical activator of high-affinity iron acquisition systems in *A. fumigatus* including the siderophore system and reductive iron assimilation [45].

**Figure 11:**
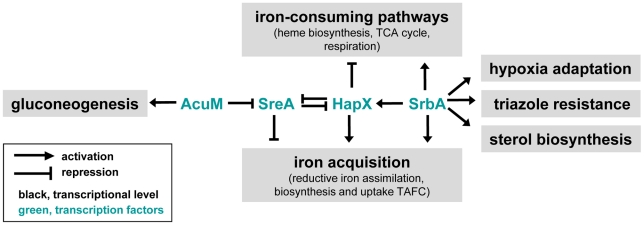
Model for relationships between the transcriptional regulators SrbA, SreA, HapX, and AcuM and their roles in iron acquisition and ergosterol biosynthesis.

Figure 11 depicts a proposed model linking SreA, HapX, and SrbA in regulation of iron acquisition and ergosterol biosynthesis. Clearly, additional studies are needed to definitively define the relationship between these three important transcriptional regulators of iron homeostasis. Future studies will also seek to incorporate AcuM into our model. Importantly, in response to hypoxia, our microarray data did not detect transcript changes in either HapX or AcuM in the SrbA null mutant. However, in iron depleted conditions, HapX transcript was clearly reduced in Δ*srbA*, which indicates that SrbA may directly or indirectly regulate HapX transcript levels under these conditions. Deletion of HapX in Δ*srbA* did increase the magnitude of reduction in siderophore levels, further suggesting a possible link between these two transcription factors that remains to be fully elucidated.

Defining a regulatory role for SrbA in iron acquisition is consistent with previous reports in other organisms that have linked SREBPs with regulation of sterol biosynthesis and adaptation to hypoxia. Previous studies have suggested a tight link between iron, oxygen and ergosterol biosynthesis in response to hypoxia in yeast. For example, in the model yeast *S. cerevisiae*, low iron conditions decrease the activity of the C4-sterol demethylase Erg25, and moreover, sterol synthesis in this organism requires heme [46]. While *S. cerevisiae* lacks an SREBP ortholog, *S. pombe and C. neoformans* Sre1 and *A. fumigatus* SrbA SREBPs appear to be key regulators of Erg25 and sterol biosynthesis [19], [30], [47] (Figure 1B, Figure 2, and Figure 5). Our *A. fumigatus* hypoxia transcriptome profiling data are also in agreement with similar studies in *S. pombe* and *C. neoformans* that demonstrate an increase in transcripts associated with heme, sterol biosynthesis, and iron uptake in response to hypoxia [17], [42], [48]. A recent proteomic analysis of *A. fumigatus* grown in a chemostat culture under hypoxia demonstrated that the cellular contents of heme and iron substantially increase in these conditions [49]. Thus, taken together, our results here and the results of prior seminal studies in yeast establish a tight link between iron, oxygen, ergosterol biosynthesis and fungal responses to hypoxia, which are mediated in part by SREBPs.

Further support for this conclusion comes from our results demonstrating that addition of high iron concentrations to Δ*srbA*, or derepression of iron uptake by simulataneous deletion of SreA, is able to partially rescue the triazole susceptibility and hypoxia growth phenotypes of this fungal SREBP null mutant. As a major goal of our study was to better understand the mechanisms behind the clinically relevant antifungal drug and virulence phenotypes of the SrbA null mutant, these results are particularly significant. An important question is how increased iron availability rescues these important Δ*srbA* phenotypes. To this end, the observed increase in total ergosterol levels in Δ*srbA* and Δ*srbA*Δ*sreA* strains in high iron conditions suggest a direct SREBP mediated link between cellular iron levels and ergosterol biosynthesis. This was also reflected in the SrbA dependent decrease in *erg11A* (*cyp51A*) transcript levels that could also be partially rescued by high iron. This result may explain, at least partially, the restoration of fluconazole resistance and hypoxia growth of Δ*srbA* under high iron conditions. It is important to note that *A. fumigatus* contains 2 functional 14α-demethylases (Erg11A/Cyp51A and Erg11B/Cyp51B) [50], [51]. Loss of Erg11A but not Erg11B function results in increased fluconazole susceptibility. Moreover, intriguingly, it was recently observed that fluconazole preferentially binds Erg11B, thus likely explaining *A. fumigatus*'s inherent resistance to fluconazole [52].

In *Candida albicans*, a link between iron availability and fluconazole susceptibility has been suggested [53]. The authors observed a 30% reduction in ergosterol levels in low iron conditions and speculate that the increased fluconazole susceptibility in these conditions was due to a subsequent increase in membrane fluidity [53]. Thus, the partial rescue of fluconazole resistance in *A. fumigatus* Δ*srbA* by high iron may in part be due to a reduction of membrane fluidity. In support of this hypothesis, ergosterol levels in Δ*srbA* are approximately 50% less than wild-type, and increases in exogenous iron partially rescue this defect, which in theory could decrease membrane fluidity. How iron increases ergosterol levels is unknown, but it could be argued that the increased iron levels improve the efficiency of ergosterol biosynthetic enzymes whose levels appear to be reduced in the absence of SrbA. Both Erg11A and Erg25 require iron as a co-factor for their enzymatic functions. Thus, the observed increases in *erg11A* transcript levels in the presence of high iron could be due to a positive feedback loop activated by an increase in sterol intermediates that result from increased enzyme efficiency.

With regard to the potential link between hypoxia growth and fungal virulence, high iron conditions or concomitant inactivation of SreA could partially rescue the hypoxia growth phenotype of Δ*srbA*, but not fungal virulence (Figure 9). Derepression of siderophore biosynthesis and iron uptake in Δ*srbA* was not dramatic enough to rescue the virulence defect of Δ*srbA* leaving the exact mechanism of SrbA's role in fungal virulence undefined. However, given that iron is a major limiting micronutrient *in vivo*, and that the effect of high iron on Δ*srbA* growth was modest, this result is not surprising. As *A. fumigatus* mutants that lack siderophore biosynthesis also have attenuated virulence *in vivo*, it seems clear that SrbA's role in siderophore biosynthesis and iron uptake is at least partially related to the inability of Δ*srbA* to cause lethal disease. Attempts to fully restore hypoxia growth of Δ*srbA* via genetic manipulation of iron homeostasis and ergosterol biosynthesis pathways are currently underway.

In conclusion, our data suggest a new role for SREBPs in linking hypoxia adaptation, iron acquisition and ergosterol biosynthesis in fungi. We believe that untangling the web of SrbA regulated effectors will lead to a better understanding of SrbA's role in fungal pathogenesis and triazole drug susceptibility, which should provide a clearer picture regarding the potential of fungal SREBP modulation as a clinical therapeutic for human disesases caused by fungi. Thus, future studies will continue to seek to elucidate the genetic regulatory network mediated by SrbA in *A. fumigatus* and its relationship to fungal virulence and triazole drug interactions. It might also be intriguing to determine the extent to which SREBPs in other eukaryotic organisms are involved in iron homeostasis mechanisms and how this potential regulation is linked with sterol biosynthesis homeostasis especially in hypoxic stress environments.

## Materials and Methods

### Fungal strains and growth conditions

*A. fumigatus* strains were grown at 37°C in *Aspergillus* minimal medium (AMM) according to Pontecorvo *et al.* [54] containing 1% glucose as carbon source and 20 mM glutamine as nitrogen source or glucose minimal medium (GMM) with 1% glucose as carbon source as previously described [19]. Iron-repleted media (+Fe) were supplemented with 30 µM FeSO_4_ and high iron media contained 1.5 mM, 3.0 mM, 5 mM or 10 mM FeSO_4_, respectively. Media used and concentrations of key elements are denoted according to the respective experiments. For iron depleted conditions (−Fe) addition of iron was omitted. For hypoxic conditions, 13.45 g Anero*Gen*™ was used or an INVIVO_2_ Hypoxia Chamber (Ruskinn) set at 1% O_2_, 5% CO_2_, 94% N_2_. For liquid growth assays, 10^8^ conidia were inoculated in 200 ml minimal medium.

### Transcriptome analysis

#### Nucleic acid extraction

Tissue was resuspended in 1 ml of Trizol reagent followed by a five minute incubation at RT. 0.2 volumes of chloroform was added, followed by a short vortex at low speed followed by 2 minute incubation at RT. Tubes were centrifuged at 16,000×g for 15 min at 4°C. The clear upper layer was pipeted into new tube and an equal volume of 80% EtOH was added. Samples were mixed well and applied to RNeasy spin column (Qiagen RNA kit) following manufacturer's instructions. RNA was eluted with 100 µL of RNase free water. Column incubated for one minute before centrifugation to elute maximum amount of RNA.

#### cDNA preparation and probe labeling

10 µg of total RNA was used for cDNA synthesis using SuperScriptIII (Invitrogen), following the “Microbial RNA aminoallyl labeling for microarrays” (SOP# M007 Rev. 2) protocol detailed at http://pfgrc.jcvi.org/index.php/microarray/protocols.html. Briefly, samples were RNaseH treated and the cDNA concentration was checked on a Nanodrop 1000. cDNA was purified with Qiagen QIAquick PCR purification kit. Concentration was rechecked with Nanodrop and samples were dried completely with Eppendorf speed-vac. Pellet was resuspended with 4.5 µL of fresh 0.1 M Na_2_CO_3_ buffer solution. 4.5 µL of Cy3 or Cy5 dye was added to appropriate tubes, and coupling was completed. Uncoupled dye was removed with NaOAc-modified QIAquick PCR cleanup. Dye ratio was calculated with Nanodrop. The two differentially labeled probes (Cy3 vs. Cy5) that were hybridized to the same microarray slide are mixed with equal cDNA volumes. The Cy3/Cy5 probe mixture was dried to completion in Eppendorf speed-vac. Resulting pellet was suspended in 10 µL of dH_2_O.

#### Microarray hybridization

Spotted arrays (*Aspergillus fumigatus* Af293, version 3) from the pathogen functional genomics resource center at JCVI were used for the entire experiment (http://pfgrc.jcvi.org/index.php/microarray/array_description/aspergillus_fumigatus/version3.html). The protocol “Microbial Hybridization of labeled probes” (SOP# M008 Rev 2.1) can be found at: http://pfgrc.jcvi.org/index.php/microarray/protocols.html. Briefly, the slides were soaked in sterile-filtered pre-hybridization solution (5×SSC, 1%BSA, 0.2%SDS) for two hours, washed and dried by centrifugation in mini slide spinner (LabNet) prior to hybridization. 45 µL of hybridization mixture (50% formamide, 5×SSC, 0.1%SDS, 0.001 M DTT) and 6 µL of salmon sperm DNA were added to probe. Lifter slip (Erie Scientific) was washed in 100% EtOH and dried. The slide and lifter slip were placed in hybridization chamber (Corning) and 60 µL of probe mixture was pipeted under lifter slip. Chambers were sealed and incubated in 42°C water bath for 18 hours. Slides were washed twice in low stringency buffer (2×SSC, 0.2% SDS, 0.02 M DTT), twice in medium stringency buffer (0.1× SSC, 0.1% SDS, 0.02 M DTT), twice in high stringency buffer (0.1× SSC, 0.02 M DTT), and a final wash with dH2O and 0.02 M DTT. Slides were dried completely in mini slide spinner.

#### Image processing

Slides were scanned with GenePix 4000B dual wavelength scanner (Axon Instruments, Molecular Devices Co.), adjusting PMT gain ratio to ∼1.0, 100% laser power, and pixel size of 10. The resulting images were checked by eye for misaligned regions or false signals using GenePixPro 6.0 (Axon Instruments, Molecular Devices Co.). A GenePix report file was generated with raw data reads for each spot. These raw files are available at EMBL MIAMExpress website (http://www.ebi.ac.uk/arrayexpress/), accession number E-MEXP-3172.

#### Data processing

Data were processed using TM4 software and protocol recommendations for microarray analysis (http://www.tm4.org/). Briefly, GenePix files were converted to MeV files using ExpressConverter 2.1. MeV files were analyzed with MIDAS 2.21 to normalize data, according to the recommended settings from TM4. Flip-dye pairs were read into MIDAS using a generous setting for one bad channel, and A and B channel flag check selected. LOWESS was used to minimize effect of intensity dependent bias, with default settings. Standard deviation regularization was used to minimize the effect of slide printing errors, with Cy3 as the reference. Flip-dye pairs were then checked for consistency and merged into a single MeV file. Biological replicates were then averaged to a single value for each gene and timepoint. This file is also available at MIAMExpress (http://www.ebi.ac.uk/arrayexpress/). Pathway analysis was then completed using gene set enrichment analysis (GSEA).

#### Chromatin immunoprecipitation and ChIP-qPCR

ChIP was performed after four hours exposure to hypoxia using methods in Kim *et al.* 2010 [55]. Briefly, cells were exposed to 1% formaldehyde to crosslink proteins to DNA, nuclei were isolated with nuclei isolation kit (Sigma) and then lysed and resulting DNA sonicated to 400–700 bp fragments. ChIP was performed with 1 µg of polyclonal antibody to SrbA (amino acids 1–275) on Protein A DynaMag beads (Invitrogen), in both wild-type and Δ*srbA* strains. Negative control for ChIP was the IgG mouse antibody (Invitrogen). DNA quantity was assessed with Qubit 2.0 Fluorometer, using the high sensitivity dsDNA assay (Invitrogen). All ChIP samples were diluted 10 fold for PCR. 1 µl of template was used in a 10 µl total volume reaction using Promega 2× GoTaq qPCR master mix and 0.4 µM of each primer. Realtime PCR was performed with 40 cycles of 95°C for 15 s and 60°C for 30 s on Mastercycler ep *realplex* PCR machine. PCR was performed in duplicate for two separate ChIP experiments using primers designed for regions identified as enriched in preliminary ChIP-SEQ analysis (Barker et al. unpublished). Three genes were chosen from this analysis as positive for enrichment (*sit1*, *erg11A* and e*rg25A*) and one was chosen as negative for enrichment (*sidA*). Percent input method was used to calculate the signal of enrichment of the promoter region for each gene (http://cshprotocols.cshlp.org/cgi/content/full/2009/9/pdb.prot5279 and Invitrogen website). Briefly, 100*(2^(InputCt-ChIPCt)^) was calculated for each reaction and the average and standard deviation calculated from these values. No correction for adjusted input was necessary as both templates were diluted equally prior to PCR. Oligonucleotides used for ChIP analysis are provided in Datasets S1 and S2.

#### Quantitative real-time PCR

qRT-PCR to measure transcript abundance was performed as we have previously described [56], [57].

### Manipulation of nucleic acids and Northern analysis

Standard molecular techniques were performed using the pGEM-T vector system (Promega) and the bacterial strain *Escherichia coli DH5α* cultivated in LB medium (1% bacto-tryptone, 0.5% yeast extract, 1% NaCl, pH 7.5) as we have previously described [2], [15]. RNA was isolated using TRI reagent (Sigma-Aldrich). 10 µg of total RNA were used for electrophoresis on 1.2% agarose −2.2 M formaldehyde gels and blotted onto Hybond N membranes (Amersham Biosciences). Probes used in this study were generated by PCR with the digoxigenin labeling system (Roche Molecular Biochemicals); Oligonucleotides used for Northern analysis are provided in Dataset S3.

### Deletion of *hapX* and *sreA* in Δ*srbA* and CEA10

Deletion of *sreA* and *hapX* in CEA10 or Δ*srbA* backgrounds was carried out as described previously for *A. fumigatus* ATCC46645 using the same deletion constructs [2], [15].

Oligonucleotides used for deletions are provided in Dataset S4.

### Analysis of siderophore production and free amino acid pools

Isolation and analysis of extra- and intracellular siderophores from culture supernatants and cellular extracts, respectively, was carried out as described previously [58], [59]. Quantification of free amino acid pools was carried out as described previously [60].

### Susceptibility testing to fluconazole and total ergosterol content measurements

E-test strips (AB bioMérieux), plastic strips impregnated with a gradient of fluconazole were used per manufacturers' instructions. Each strip was placed onto a AMM agar plate without iron or supplemented with 30 µM or 10 mM FeSO_4_ containing a lawn of conidia of the respective strain and growth inhibition was measured after 24 and 48 h by direct observation of the plates at 37°C. No difference in results was observed between 24 and 48 hours. Total ergosterol content was measured as previously described [61]. Total ergosterol content results are the mean and standard deviation from 2 biological replicates with 6 total technical replicates for each strain.

### Murine virulence assay of invasive pulmonary aspergillosis

6 to 8 weeks old outbred CD-1 mice were immunosuppressed with intraperitoneal (i.p.) injections of cyclophosphamide at 150 mg/kg 2 days prior to inoculation and 40 mg/kg Kenalog injected subcutaneously (s.c.) 1 day prior to inoculation. Repeat injections were given on day 3 post inoculation with cyclophosphamide (150 mg/kg i.p.) and on day 6 post inoculation with Kenalog (40 mg/kg s.c.). Mice were housed six per cage and had access to food and water ad libitum. Twelve mice per *A. fumigatus* strain (CEA10 and Δ*srbA*Δ*sreA*) were inoculated intranasally with 1×10^6^ conidia/40 µl following brief isofluorane inhalation. Mock control mice were inoculated with sterile 0.01% Tween 80. Mice were monitored twice daily over a time period of 14 days. Any animals showing distress were immediately sacrificed and recorded as deaths within 24 hrs. No mock infected animals perished during the time course of the experiment. All experiments were approved by the Montana State University IACUC and adhere to NIH policies on animal welfare.

### Ethics statement

This study was carried out in strict accordance with the recommendations in the Guide for the Care and Use of Laboratory Animals of the National Institutes of Health. The animal experimental protocol was approved by the Institutional Animal Care and Use Committee (IACUC) at Montana State University (Federal-Wide Assurance Number: A3637-01).

### Histopathology

For histopathology, five CD-1 mice per *A. fumigatus* strain (CEA10, Δ*srbA*, Δ*srbA*Δ*sreA*) were immunosuppressed and inoculated as described above. On day 4 post *A. fumigatus* challenge, mice were sacrificed by pentobarbital anesthesia (100 µg/g body weight) followed by exsanguinations. Lungs were removed immediately, fixed in 10% phosphate-buffered formalin, embedded in paraffin, sectioned at 5 µm, and stained with hematoxylin, and eosin (H&E) or Gomori methenamine silver (GMS) by using standard histological techniques. Microscopic examinations were performed on a Nikon Eclipse 80i microscope and imaging system (Nikon Instruments Inc., Melville, NY, USA).

## Supporting Information

Figure S1 Gene set enrichment analysis for gene ontology molecular function. Heat map representing the results of the gene set enrichment analysis on the gene ontology term molecular function from the wild-type and Δ*srbA* hypoxia microarray experiment. The upper left of each square depicts upregulated mRNAs (those expressed higher in Δ*srbA*) while the lower right of each square represents downregulated mRNAs (those expressed higher in the wild-type). Color denotes the level of significance as depicted in the bar above the GO terms. The more yellow the square, the more significant the association with that GO term.
(TIF)

Figure S2 Gene set enrichment analysis for gene ontology Cellular Component. Heat map representing the results of the gene set enrichment analysis on the gene ontology term Cellular Component from the wild-type and Δ*srbA* hypoxia microarray experiment. The upper left of each square depicts upregulated mRNAs (those expressed higher in Δ*srbA*) while the lower right of each square represents downregulated mRNAs (those expressed higher in the wild-type). Color denotes the level of significance as depicted in the bar above the GO terms. The more yellow the square, the more significant the association with that GO term.
(TIF)

Figure S3 Gene set enrichment analysis for gene ontology biological process. Heat map representing the results of the gene set enrichment analysis on the gene ontology term biological process from the wild-type and Δ*srbA* hypoxia microarray experiment. The upper left of each square depicts upregulated mRNAs (those expressed higher in Δ*srbA*) while the lower right of each square represents downregulated mRNAs (those expressed higher in the wild-type). Color denotes the level of significance as depicted in the bar above the GO terms. The more yellow the square, the more significant the association with that GO term.
(TIF)

Figure S4 Increased iron availability and/or inactivation of SreA improve resistance of Δ*srbA* against cobalt chloride. 10^3^ conidia of each strain were point-inoculated on AMM agar plates containing different iron concentrations (−Fe; +Fe, 30 µM; hFe, 1.5 mM; hhFe, 3.0 mM) or the iron chelator BPS (−Fe, 100 µM BPS) in the presence or absence of 0.6 mM CoCl_2_ and incubated for 48 h at 37°C.
(TIF)

Table S1 Microarray results, Log_2_ ratios and fold changes for mRNAs downregulated in Δ*srbA* at 1 hour post exposure to hypoxia.
(XLS)

Table S2 Microarray results, Log_2_ ratios and fold changes for mRNAs upregulated in Δ*srbA* at 1 hour post exposure to hypoxia.
(XLS)

Table S3 Microarray results, Log_2_ ratios and fold changes for mRNAs downregulated in Δ*srbA* at 2 hours post exposure to hypoxia.
(XLS)

Table S4 Microarray results, Log_2_ ratios and fold changes for mRNAs upregulated in Δ*srbA* at 2 hours post exposure to hypoxia.
(XLS)

Table S5 Microarray results, Log_2_ ratios and fold changes for mRNAs downregulated in Δ*srbA* at 4 hours post exposure to hypoxia.
(XLS)

Table S6 Microarray results, Log_2_ ratios and fold changes for mRNAs upregulated in Δ*srbA* at 4 hours post exposure to hypoxia.
(XLS)

Table S7 P-values for Gene set enrichment analysis of gene expression data for gene ontology molecular function.
(XLS)

Table S8 P-values for Gene set enrichment analysis of gene expression data for gene ontology cellular component.
(XLS)

Table S9 P-values for Gene set enrichment analysis of gene expression data for gene ontology biological process.
(XLS)

Dataset S1 Oligonucleotides used in this study for ChIP promoter enrichment.
(DOCX)

Dataset S2 Oligonucleotides sequences used in Realtime RT-PCR.
(DOCX)

Dataset S3 Oligonucleotides used in this study for Northern-blot probes.
(DOCX)

Dataset S4 Oligonucleotides used for generation of deletion strains.
(DOCX)
