# Supplementary figures and images for: SREBP Coordinates Iron and Ergosterol Homeostasis to Mediate Triazole Drug and Hypoxia Responses in the Human Fungal Pathogen Aspergillus fumigatus
Source: PLoS Genet. 2011 Dec 1;7(12):e1002374. doi: 10.1371/journal.pgen.1002374 (PMC3228822; doi:10.1371/journal.pgen.1002374)

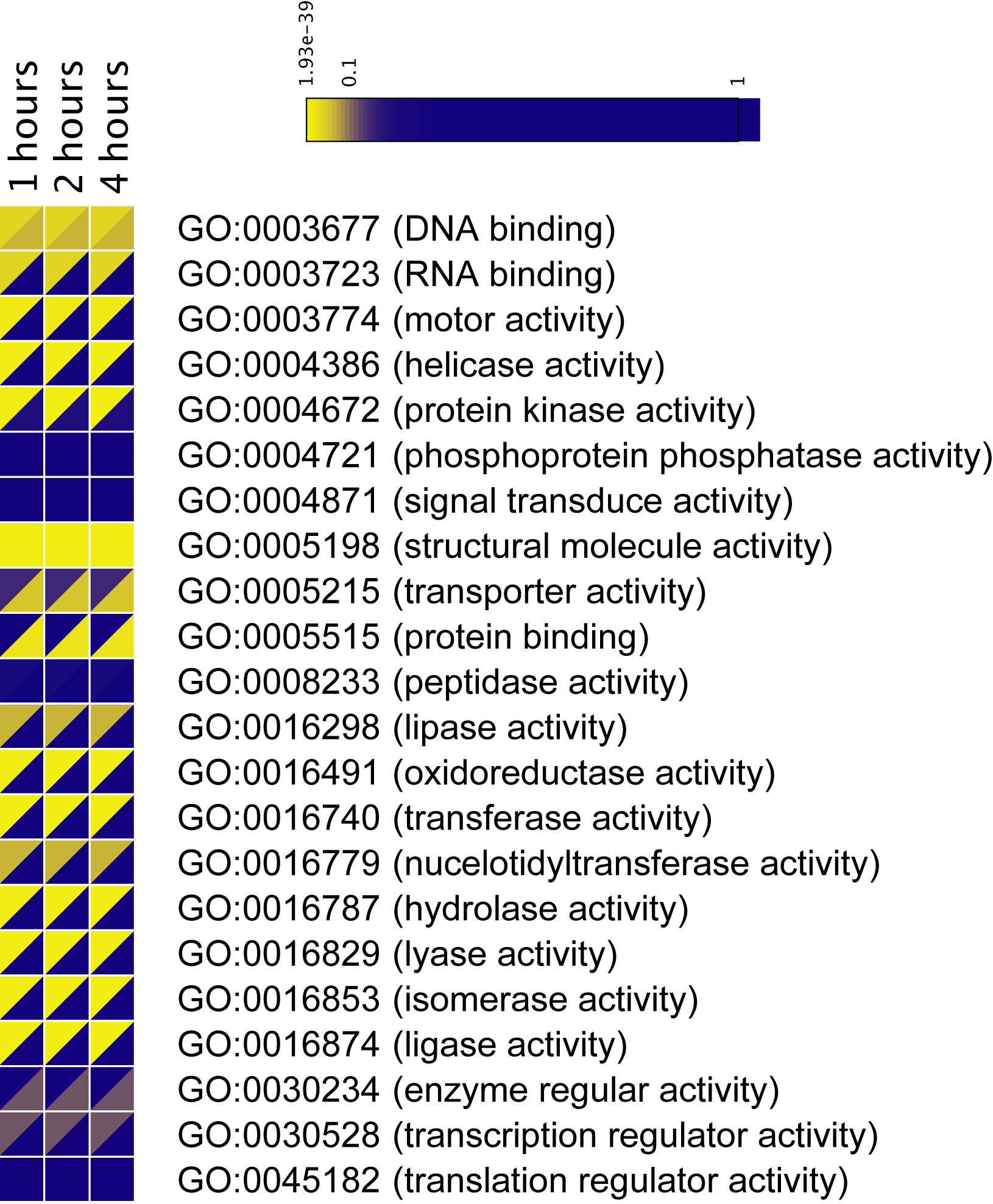

Supplement: Figure S1 — Gene set enrichment analysis for gene ontology molecular function. Heat map representing the results of the gene set enrichment analysis on the gene ontology term molecular function from the wild-type and ΔsrbA hypoxia microarray experiment. The upper left of each square depicts upregulated mRNAs (those expressed higher in ΔsrbA) while the lower right of each square represents downregulated mRNAs (those expressed higher in the wild-type). Color denotes the level of significance as depicted in the bar above the GO terms. The more yellow the square, the more significant the association with that GO term. (TIF) [file pgen.1002374.s001.tif]

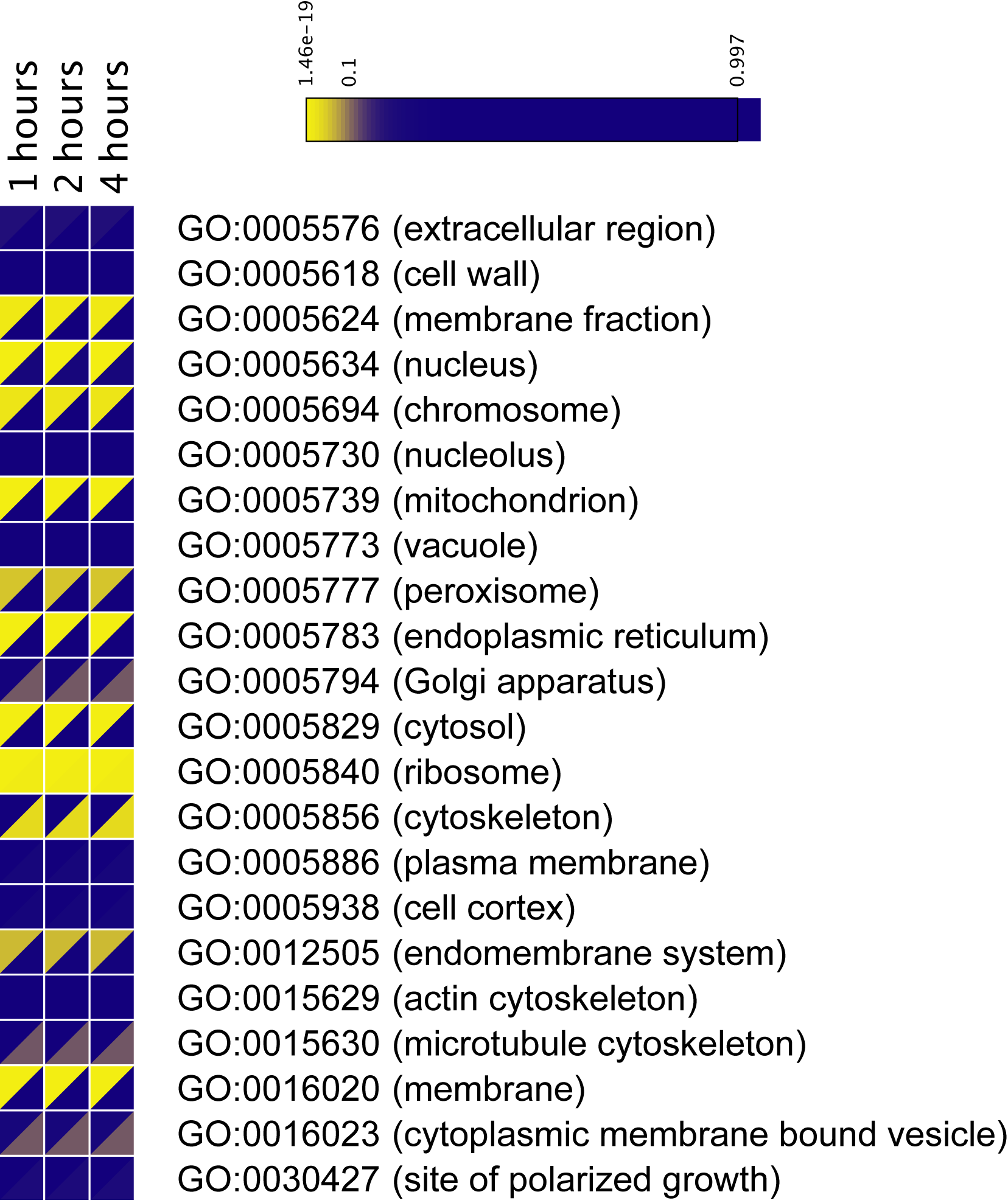

Supplement: Figure S2 — Gene set enrichment analysis for gene ontology Cellular Component. Heat map representing the results of the gene set enrichment analysis on the gene ontology term Cellular Component from the wild-type and ΔsrbA hypoxia microarray experiment. The upper left of each square depicts upregulated mRNAs (those expressed higher in ΔsrbA) while the lower right of each square represents downregulated mRNAs (those expressed higher in the wild-type). Color denotes the level of significance as depicted in the bar above the GO terms. The more yellow the square, the more significant the association with that GO term. (TIF) [file pgen.1002374.s002.tif]

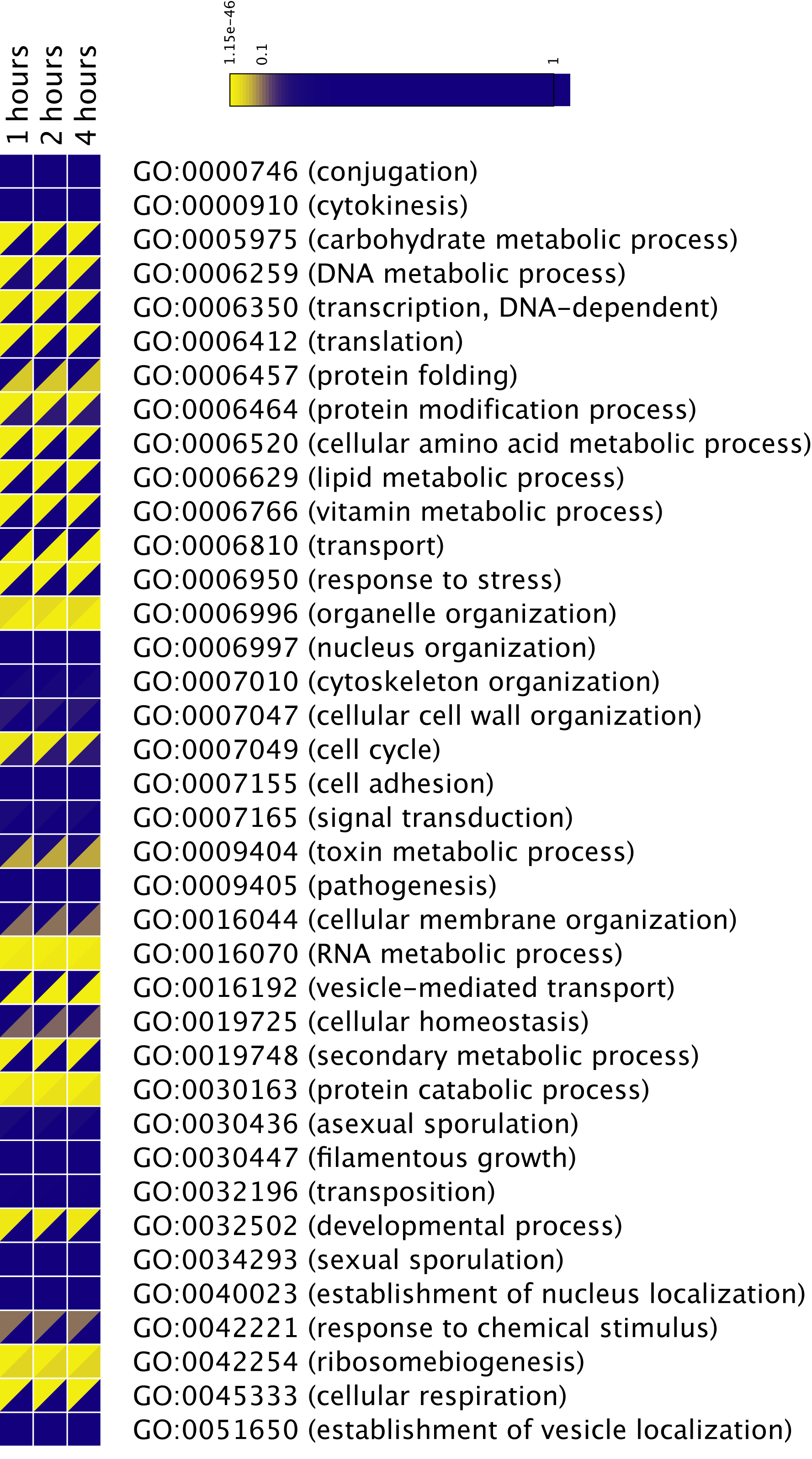

Supplement: Figure S3 — Gene set enrichment analysis for gene ontology biological process. Heat map representing the results of the gene set enrichment analysis on the gene ontology term biological process from the wild-type and ΔsrbA hypoxia microarray experiment. The upper left of each square depicts upregulated mRNAs (those expressed higher in ΔsrbA) while the lower right of each square represents downregulated mRNAs (those expressed higher in the wild-type). Color denotes the level of significance as depicted in the bar above the GO terms. The more yellow the square, the more significant the association with that GO term. (TIF) [file pgen.1002374.s003.tif]

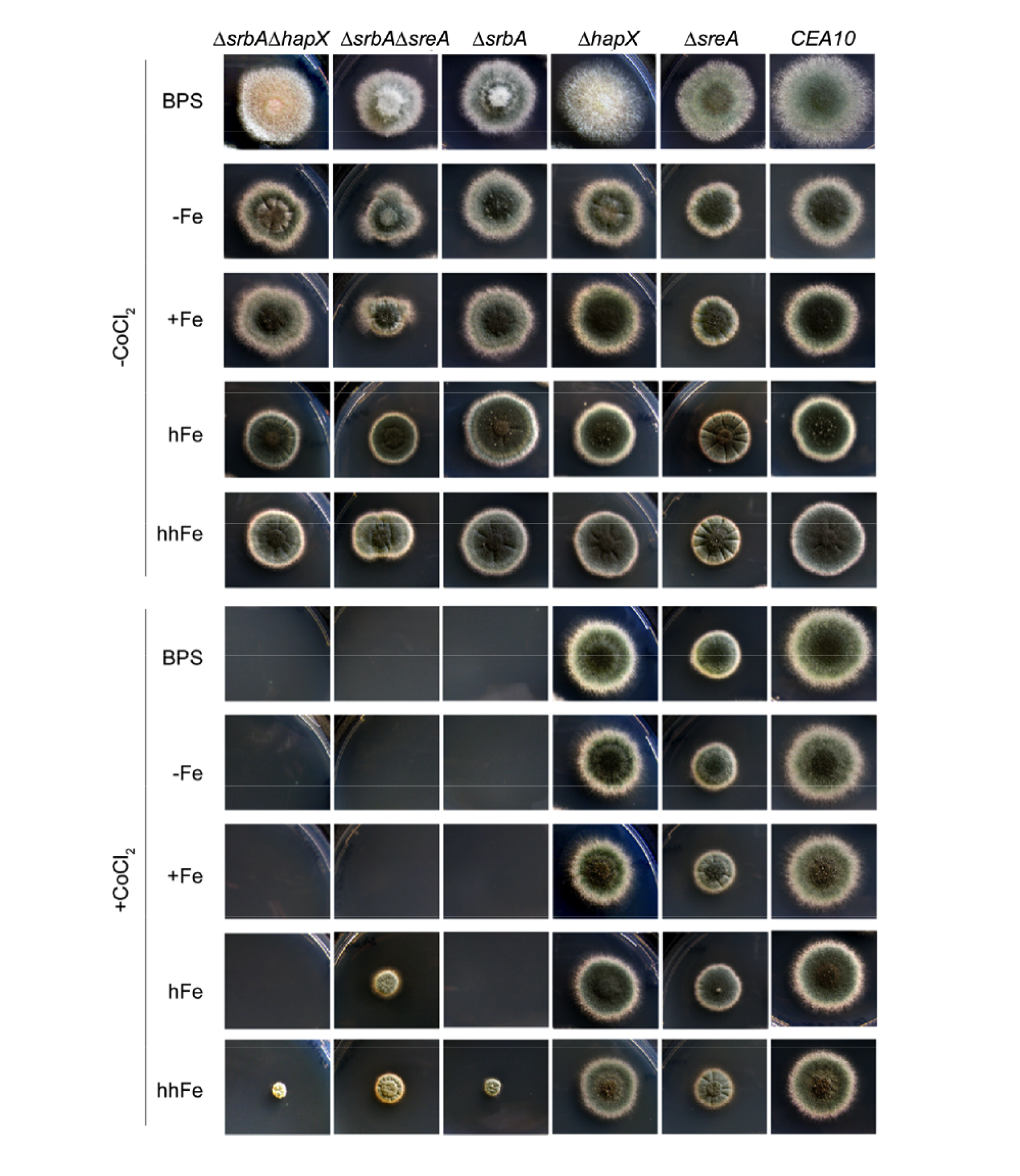

Supplement: Figure S4 — Increased iron availability and/or inactivation of SreA improve resistance of ΔsrbA against cobalt chloride. 103 conidia of each strain were point-inoculated on AMM agar plates containing different iron concentrations (−Fe; +Fe, 30 µM; hFe, 1.5 mM; hhFe, 3.0 mM) or the iron chelator BPS (−Fe, 100 µM BPS) in the presence or absence of 0.6 mM CoCl2 and incubated for 48 h at 37°C. (TIF) [file pgen.1002374.s004.tif]
