# Supplementary material for: SREBP Coordinates Iron and Ergosterol Homeostasis to Mediate Triazole Drug and Hypoxia Responses in the Human Fungal Pathogen Aspergillus fumigatus
Source: PLoS Genet. 2011 Dec 1;7(12):e1002374. doi: 10.1371/journal.pgen.1002374 (PMC3228822; doi:10.1371/journal.pgen.1002374)
Supplement: Dataset S1 — Oligonucleotides used in this study for ChIP promoter enrichment. (DOCX) [file pgen.1002374.s014.docx]

**Oligonucleotides used in this study for ChIP promoter enrichment.**

| **Gene** | **Oligonucleotides** | **Sequence** |
| --- | --- | --- |
| *erg11A,* AFUA_ 4G06890 | erg11A (cyp51A) 3’ | AACTCTGGAGTGGTGCTGCGATTA |
|  | erg11A (cyp51A) 5’ | GCAGCATTCTGAAACACGTGCGTA |
| *erg25A,* AFUA_ 8G02440 | erg25A 3’  erg25A 5’ | TCAAGTTACCCGGGATGGCATGAT TCGCTTGTCCCTGCATAGCAACTA |
| *sit1,* AFUA_7G06060 | sit1 3’ | ACCCAAGTGTACGGACAAGGTCAA |
|  | sit1 5’ | AGTCGCACTGTCACACAAAGGTCA |
| *sidA,* AFUA_2G07680 | sidA 3’ | ACCGGTAGGAATCTTCGTCGAGCA |
|  | sidA 5’ | AATGTGTTCTGGCTTGGGATTGGG |
