# Supplementary material for: SREBP Coordinates Iron and Ergosterol Homeostasis to Mediate Triazole Drug and Hypoxia Responses in the Human Fungal Pathogen Aspergillus fumigatus
Source: PLoS Genet. 2011 Dec 1;7(12):e1002374. doi: 10.1371/journal.pgen.1002374 (PMC3228822; doi:10.1371/journal.pgen.1002374)
Supplement: Dataset S2 — Oligonucleotides sequences used in Realtime RT-PCR. (DOCX) [file pgen.1002374.s015.docx]

**Primer sequences used in Realtime RT-PCR**

Gene Oligonucleotide Sequence

|  |
| --- |

*sit1* AFUA_7G06060 sit1 5’ TGAAGGCGAAGGGATTGGCATAGA

sit1 3’ TTCAGGTCGCCTCCAAGCATAAGT

|  |
| --- |

*fetC,* AFUA_5G03790 fetC 5’ TGAGGACAATCGGGTGAGTGTTGT

fetC 3’ TATGTCATGCCCAAGGTTCCGACT

|  |
| --- |

*sidA,* AFUA_2G07680 sidA 5’ CCGCATTGCAGAATCCCGCATAAT

sidA 3’ CTTACATTGCCGTTCTGGGCA

|  |
| --- |

*ftrA,* AFUA_4G14640 ftrA 5’ AGCAACCAGGTAGAGGATGCAAGT

frtA 3’ TGTTCATTGGAGGCGTCAGTCTCA

|  |
| --- |

*erg11A,* AFUA_4G06890 erg11A 5’ AAGGAGCAGGAGAACGACAAGGTT

erg11A 3’ AGCACCAAACGGAAGATAGGGACT

|  |
| --- |

*egrg11B,* AFUA_7G03740 erg11B 5’ ATTTGCACGCGAAGGTCATCAAGG

erg11B 3’ AGTACCATCCACAGCCATGGGATT

|  |
| --- |

*erg25A,* AFUA_8G02440 erg25A 5’ TCCAGGCTATCGATTCACATTCGG

erg25A 3’ AACCGTACCCATCAACACATCCCA

|  |
| --- |

*srbA,* AFUA_2G01260 srbA 5’ ATCCATTGGTTTAATCGCCTGCCC

srbA 3’ AAGGTAGTCGATGTCTGACGCCAA

|  |
| --- |

*tub2,* AFUA_7G00250 β-tubulin, 5' ATAATGTTCAGACCGCCCTCTGCT

β-tubulin, 3' GACGGATGTGGAATTGCCCACAAA

|  |
| --- |

*tef1,* AFUA_1G06390 TefA 5’ GTGACTCCAAGAACGATCCC

TefA 3’ AGAACTTGCAAGCAATGTGG
