# Supplementary material for: SREBP Coordinates Iron and Ergosterol Homeostasis to Mediate Triazole Drug and Hypoxia Responses in the Human Fungal Pathogen Aspergillus fumigatus
Source: PLoS Genet. 2011 Dec 1;7(12):e1002374. doi: 10.1371/journal.pgen.1002374 (PMC3228822; doi:10.1371/journal.pgen.1002374)
Supplement: Dataset S3 — Oligonucleotides used in this study for Northern-blot probes. (DOCX) [file pgen.1002374.s016.docx]

**Oligonucleotides used in this study for Northern-blot probes.**

| **Gene** | **Oligonucleotides** | | **Sequence** |
| --- | --- | --- | --- |
| *hapX,* AFUA_5G03920 | oAfhapX-seq1 | | TCG GTG GAA AGA AGT GCC |
|  | oAfhapX-seq2 | | CGA GTC CGT TTG GGT ATC |
| *sreA,* AFUA_5G11260 | osreA-f  osreA-r | | CTC AGT ACG ATC GCT TCC  GTT GGA CG AGT AGG TAGC |
| *mirB,* AFUA_3G03810 | omirB1me | | AAG CCG AGA AAA AGG GGG |
|  | omirB2me | | AAC CCA GAT GAA GCC CAG |
| *sidA,* AFUA_2G07680 | Afomo1-f | | TAT TCT TAT TGA TCA GAA TCT GTT GAA CGG AAG |
|  | Afomo2-r | | TTT AAA TCT AAG CTT TTA CAG CAT GGC TCG TAG |
| *sidF,* AFUA_3G03400 | osidF-1f | CCT CAT CCC TAT CTC ACC | |
|  | osidF-2r | AgT TTT gAg CgA gAg ggg | |
| *ftrA,* AFUA_4G14640 | oftrAff | GGG ACA AGA GCA AGA TGC | |
|  | oftrAfr | CCC AGT AGA GGA TGC AAG | |
| *srbA,* AFUA_2G01260 | osrbA-f | TCA ACG GCA AAA GGG CTG | |
|  | osrbA-r | CTC GCC TCG AAA TAC CTG | |
| *hem13,* AFUA_1G07480 | ocopox1 | AAC GCT CCG ATG AAC ACC | |
|  | ocopox2 | GGT CTC TTG AGG GTT CTG | |
| erg3, AFUA_2G00320 | erg3-f | ATG CGC TTC TTC TGC CAG | |
|  | erg3-r | AAC TTG CCG CTG GAT CTC | |
| *erg25,* AFUA_8G02440 | oerg25-1 | TTC GTC CTA TCC GCA GTC | |
|  | oerg25-2 | TCT CTT CTC CCT CGC TTC | |
| *acoA,* AFUA_6G12930 | acoA-f | CAG CGT CCT CTC ACA TAC | |
|  | acoA-r | GCA AGA ACC GAT CAG ACC | |
