# Supplementary material for: SREBP Coordinates Iron and Ergosterol Homeostasis to Mediate Triazole Drug and Hypoxia Responses in the Human Fungal Pathogen Aspergillus fumigatus
Source: PLoS Genet. 2011 Dec 1;7(12):e1002374. doi: 10.1371/journal.pgen.1002374 (PMC3228822; doi:10.1371/journal.pgen.1002374)
Supplement: Dataset S4 — Oligonucleotides used for generation of deletion strains. (DOCX) [file pgen.1002374.s017.docx]

**Oligonucleotides used for generation of deletion strains.**

| **Gene** | **Oligonucleotides** | **Sequence** |
| --- | --- | --- |
|  | ohapX-1 | AGC GAC TAT AGC CGG ATG |
|  | ohapX-2 | CCT TGG GTC TTG AAG CTT GCG |
|  | ohapX-3 | TCA TCT AGA CTG CCC AAG CTT CAT ACC |
| *hapX,* AFUA_5G03920 | ohapX-4 | ATC AGA GCT GGA GAG GCA |
|  | ohapX-5 | TGG AGT TCC GAT TGG TGC |
|  | ohapX-6 | ATC CCG CTT CTT TCA CCC |
|  | osreA1 | AAC CCG CAT GTC TAA GCC |
| *sreA,* AFUA_5G11260 | osreA2 | CAC GCA GCA CTG AAT CAC |
| *prtA* (plasmid pSK275) | oprtA1 | GAG GAC CTG GAC AAG TAC |
|  | optrA2 | CAT CGT GAC CAG TGG TAC |
| *hph,* (plasmid pHPH) | Dig hph 1 | CCA TAC TCC ATC CTT CCC |
|  | Dig hph 2 | TTC TGC GGG CGA TTT GTG |
